# Engineering Liposomes and Polymer Conjugates: A Platform for Mechanistic Complement Activation Studies and Controlled Release Applications

**DOI:** 10.1021/acsami.6c02923

**Published:** 2026-04-21

**Authors:** Kilian Hoecherl, Johannes Konrad, Christina Reiner, Simon Streif, Clemens Spitzenberg, Carola Sommer, Diana Pauly, Miriam Breunig, Antje J. Baeumner

**Affiliations:** † Institute of Analytical Chemistry, Chemo- and Biosensors, 9147 University of Regensburg, Universitätsstraße 31, 93053 Regensburg, Germany; ‡ Department of Pharmaceutical Technology, University of Regensburg, Universitätsstraße 31, 93053 Regensburg, Germany; § Experimental Ophthalmology, 9377 University of Marburg, Baldingerstraße, 35043 Marburg, Germany

**Keywords:** liposomes, controlled release, complement system, immunoassay, diagnostic assay

## Abstract

Due to their ability to encapsulate both hydrophilic and hydrophobic molecules and to allow for their controlled release, liposomes have evolved into a promising and frequently used tool in medicine, biotechnology, and bioanalysis. In this work, we designed liposome surfaces and polymer conjugates to reliably and in a controlled fashion activate the complement system and monitor its function. Specifically, the polymer conjugates were designed on protein, polysaccharide, or synthetic polymer backbones, respectively, enabling flexible coupling chemistry and size tuning. They were optimized as trigger entities for efficient stimulation of complement responses through specific surface interactions with the liposome membrane and complement proteins simultaneously. Additionally, the liposome surface chemistry was optimized to ensure the specificity of the binding and complement stimulation. Studies with human serum confirmed the applicability of the new assay principle providing in-depth understanding of complement action. Specifically, increasing the density of trigger moieties enhanced the complement activation efficiency. Complement lysis strongly relies on the physiological geometry of trigger and recognition sites and correlates with cross-linking of liposomes. Since the liposomes demonstrated high long-term stability and the trigger entities offered a range of polymer backbones, this new principle is a platform technology that will be applicable for a broad spectrum of assays, including immunoassays, in-depth investigations of complement activation and regulation, and targeted release of liposome encapsulants for drug delivery systems.

## Graphical abstract

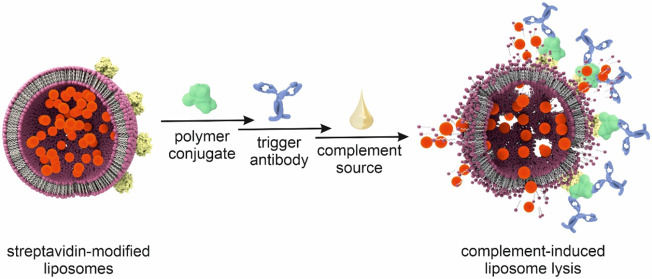

## Introduction

Liposomes are nanoscale vesicles composed of a lipid bilayer and an aqueous cavity^1^ and have evolved into a widely used tool in various fields such as drug delivery,^2^ vaccine development,^3^ cosmetics,^4^ food industry,^5^ bioanalysis and diagnostics.^6,7^ The structural features of liposomes enable the encapsulation of hydrophilic molecules in the aqueous cavity and hydrophobic substances within the lipid bilayer. Their surface can easily be modified using functionalized lipids and common conjugation strategies to tailor them toward a desired application. The chemical composition renders liposomes easily biocompatible, which has made them a well-established carrier platform in drug delivery.^2,8^ Despite many advantages, a major challenge remains unsolved, which is the spatially and temporally controlled release of encapsulated molecules, which is of utmost importance for diagnostic applications and drug delivery.

Release from conventional liposomes is—depending on the physicochemical properties of the cargo molecule—rather slow and uncontrolled. Therefore, liposomes have been designed to release their content based on environmental triggers such as pH or temperature.^9–11^ In pH-sensitive systems, liposome destabilization is induced by acidic environments in endosomes or cancer tissue through protonation of specific lipids leading to membrane fusion or leakage.^9^ Thermosensitive liposomes, on the other hand, undergo phase transitions at defined temperatures close to physiological conditions allowing localized drug release upon mild hyperthermia.^10^ While these strategies are effective, they may suffer from limitations such as premature leakage, limited control over release kinetics and the presence of a specific microenvironment, which restricts their broader applicability.^12^ Other strategies designed to trigger the release of liposome cargo molecules are based on biological, chemical or physical stimuli. Physical and chemical methods include sonication, photoirradiation, electric fields, detergents or oxidation of lipids.^13–15^ These approaches often rely on harsh or nonspecific conditions and are difficult to apply selectively *in vivo*. Biological approaches employ enzymes such as phospholipases A_2_, C, or D,^13,16^ or bacterial pore-forming toxins (e.g., α-hemolysin^17^ or cytolysin A)^18^ that disrupt lipid bilayers by cleavage of lipids or formation of transmembrane pores. Although such mechanisms can be highly effective, their use may be limited by low specificity and potential immunogenicity for therapeutic applications. Despite extensive optimizations of all these strategies over the last couple of years,^13,19,20^ they have not provided the necessary breakthrough of spatial and temporal release. Therefore, we propose a new and highly specific release strategy for molecules that have been encapsulated into liposomes. It operates under mild, physiological conditions and enables a controlled liposome lysis by exploiting the complement system as a trigger of cargo release.

The complement system is part of the innate immune system and a complex, tightly regulated network of plasma proteins, which can be divided into three distinct pathways: the classical, alternative and lectin pathway.^21^ They are activated through specific surface recognition elements such as antigen–antibody complexes or sugar patterns, which trigger a proteolytic cascade of enzymatic reactions involving the recruitment and cleavage of several complement proteins.^22^ Formation of the complement protein C5b is the final step of each activation pathway, ultimately leading to a common terminal pathway, which involves the association of C5b with complement proteins C6–9 and results in the formation of the membrane attack complex (MAC).^22^ The MAC inserts into and penetrates lipid bilayers by forming 10 nm wide pores, which has been demonstrated in previous studies using techniques such as atomic force microscopy (AFM) on supported bilayers,^23^ as well as electron microscopy and cryo-electron tomography of liposomes.^24,25^ In nature, this event leads to destruction of pathogens. In our case, the MAC is intended to be inserted into the liposomal membrane and thus will realize the release of encapsulated substances through the pores.^26^ The complement system is not only involved in a wide range of diseases^27,28^ but also needs to be considered in the context of drug and nanoparticle application, making a deep understanding of underlying mechanisms essential for biomedical research, diagnostics, and drug development.^29^ The role of the complement system in immune defense, cancer and autoimmune diseases is the current focus of numerous studies,^27,30–32^ hence the platform developed here will provide a simple and versatile tool for mechanistic and functional investigations, harnessing the biomimetic properties of liposomes and exploring their potential in drug delivery applications.

Recently, we have demonstrated that liposome lysis achieved through the complement system can be applied for diagnostics of anti-PEG or anti-SARS-CoV-2 antibodies in human sera.^33,34^ In the latter study, the platform demonstrated an excellent correlation with a clinically approved ELISA (*R* ^2^ = 0.82, Spearman *r* = 0.90), confirming its suitability for reliable quantitative analysis.^34^ The huge advantage was that the assay principle allowed for realization of a homogeneous assay format, which is more rapid and needs no washing steps compared to the conventional heterogeneous ELISA-based format.^35^ Liposomes containing the fluorophore sulforhodamine B (SRB) within their aqueous core were also applied in this study to monitor pore formation via MAC and the release of encapsulants. Here, the self-quenching property of SRB is exploited, resulting in only minimal fluorescence from intact liposomes. As soon as the MAC is inserted into the liposomal membrane, SRB exit occurs, and its fluorescence signal is taken as a measure for successful release. Both end point and time-resolved measurements are possible, which allows the study of release kinetics of SRB. Since SRB exhibits physicochemical properties similar to many clinically relevant liposomal small molecule therapeutics—including water solubility and membrane impermeability and a comparable molecular weight to drugs such as doxorubicin, methotrexate or irinotecan—it serves as a suitable model molecule for typical drug delivery cargos. Control over the liposome composition and surface chemistry as well as the liposome-to-serum ratio, the amount of complement trigger per liposome, and the complement activity of the serum itself were identified as key parameters to tune the serum stability and the general performance of the liposomes.^33,34^ In our recent studies,^33,34^ antibodies serving as analytes in the diagnostic assay were exploited as trigger entities. They had to directly bind to the liposome surface for complement activation. We now aim to advance this technology to the next level, expanding its use beyond diagnostics to include drug delivery and mechanistic studies of the complement system. Therefore, we suggest trigger entities based on different (bio)­polymeric backbone materials such as protein, synthetic polymers and polysaccharides that can serve as complement-mediating platform. The specific binding of these polymer conjugates to liposomes was enabled by their biotinylation and at the same time streptavidin functionalization of the liposome surface, which is a well-established interaction for liposome modification with biomolecules or biomaterials.^7,36–38^ This interaction has also been employed and investigated in some complement-related studies.^39–41^ However, its application in the context of multivalent polymer conjugates to modulate complement activation remains unexplored. In our study, after binding of the trigger entity to the liposome, the complement system was intended to be activated via the classical pathway. This activation was mediated by antibiotin or anti-PEG antibodies binding to the polymer conjugates. The polymer backbones allowed modification with multiple trigger moieties, resulting in a multimeric structure that promotes complement activation, as the formation of IgG oligomers is a prerequisite for efficient C1q recruitment.^42–44^ C1q serves as recognition molecule of the classical pathway and initiates the downstream complement cascade. Prior work focused on the covalent modification of the liposome surface with polymers and their effects on immunological and complement-related effects.^45–48^ Instead, here, we examined very thoroughly how the polymer backbone itself, the molecular weight and trigger density affected complement activation as well as the influence of liposome cross-linking and spatial arrangements. Previous studies have primarily focused on parameters such as lipid composition, encapsulant, vesicle size and surface design via covalent ligand attachment to investigate or exploit their effects on complement activation.^33,34,49–53^ In contrast, our study demonstrates the applicability of polymer conjugates as trigger mediators. Consequently, this tunable platform technology for the controlled release of liposome-encapsulated substances offers a wide range of potential applications, including homogeneous immunoassay formats, studies of the complement system and targeted drug release.

## Materials and Methods

### Chemicals and Consumables

The phospholipids 1,2-dipalmitoyl-*sn*-glycero-3-phosphocholine (DPPC), 1,2-dipalmitoyl-*sn*-glycero-3-phospho-(1′-*rac*-glycerol) (sodium salt) (DPPG), and the extruder set were purchased from Avanti Polar Lipids (Alabaster, AL, USA); 1,2-dipalmitoyl-*sn*-glycero-3-phosphoethanolamine-*N*-(glutaryl) (sodium salt) (*N*-glutaryl-DPPE) from Coatsome; cholesterol (≥99%, C8667), *N*-hydroxysulfo-succinimide sodium salt (sulfo-NHS) (≥98%, 56485), 2-(2-Methoxyethoxy)­ethanamine (901159; PEG-amine), ethanolamine (E9508), *n*-butylamine (8.01539), l-lysine dihydrochloride (L5751), glycine (1.04201), bovine serum albumin fraction V (BSA), polyclonal goat antibiotin antibody (B3640), monoclonal rabbit anti-PEG antibody clone RM105 (MABS1214), monoclonal mouse anti-PEG clone 6.3 (MABS1966), biotin (B4501), 4-dimethylaminopyridine (DMAP), calcium hydride, methanol, diethyl ether and dimethyl sulfoxide (DMSO), streptavidin from *Streptomyces avidinii* (85878) and Sephadex G-50 were purchased from Sigma-Aldrich/Merck (Darmstadt, Germany); sulforhodamine B (SRB) (S1307), (1-ethyl-3-(3-(dimethylamino)­propyl) carbodiimide-hydrochloride) (EDC) (PG82079), biotinylated bovine serum albumin (29130), Nunc MaxiSorp high binding microplates (437111) and the Human Complement C3a ELISA Kit (BMS2089TWO) were purchased from Thermo Fisher Scientific (Waltham, MA, USA); *n*-Octyl-β-d-glucopyranoside (OG) (≥98%, CN23), 2-(*N*-morpholino)-ethanesulfonic acid (MES) (≥99%, 4259), *N*-2-Hydroxyethylpiperazine-*N*′-2-ethanesulfonic acid (HEPES) (≥99.5%, HN78), cysteamine hydrochloride (7625), dextran 40 kDa and 10 kDa, Spectra/Por 3 dialysis membrane (MWCO 3500 Da) and Spectra-Por Float-A-Lyzer G2 (1 mL, MWCO 1000 kDa) from Carl Roth (Karlsruhe, Germany); carbonyl diimidazole (CDI) was purchased from TCI (Eschborn, Germany); biotin was purchased from BLD Pharmatech (Reinbek, Germany), 8-Arm-PEG with molecular weights 10, 20, 40 kDa was purchased hydroxyl-modified from JenKem Technology (Beijing, China). C1q inhibitor (monoclonal mouse anti-C1q85 antibody) was obtained from Sanquin (Amsterdam, Netherlands). Pooled human complement sera (batches 45270 and 38811) were purchased from Innovative Research (Novi, MI, USA). For additional information on common reagents and buffer compositions see SI (Table S1).

### Synthesis of Dextran and 8-Arm-PEG Polymer Conjugates

Exact molar masses for dextran were determined via HPLC SEC on a Phenomenex Yarra 3000 column, standardized via a dextran standards kit from PSS (Mainz, Germany). PEG was aminated in house according to Mietzner et al.^54^ Polymer modification was adapted from Bamford et al.^55^ in a Heidolph Synthesis 1 Multireactor (Schwabach Germany). First, 100 mg polymer (dextran or 8-Arm-PEG) was dissolved in approximately 3 mL of DMSO, which required some heating (50 °C) in the case of dextran. Afterward, 2 eq of DMAP with respect to biotin were added to the solution. Biotin was dissolved in an adequate amount of DMSO and activated with 1.1 eq of CDI for 30 min using CO_2_ development to estimate the progress of the reaction. The reaction was carried out overnight at 40 °C, which led to a higher coupling efficiency than room temperature. Afterward, the reaction mixture was precipitated in 4× its volume of an appropriate antisolvent (methanol for dextran, diethyl ether for PEG). PEG-biotin was purified via dialysis against Milli-Q water and lyophilized, dextran-biotin via additional precipitations (3×). All polymers were dried in vacuo overnight at 50 °C to remove any remaining solvents.

Degree of substitution (DOS) was calculated from ^1^H-NMR spectra recorded with a Bruker Avance Neo 500 MHz in D_2_O or DMSO-*d*_6_, depending on solubility. Biotin content was calculated from NMR spectra after normalizing to the dextran C1 peak (4.87 ppm) and relating to measured *M*_n_ (number-average molecular weight) from SEC.

### Liposome Preparation

Liposomes were prepared as previously described using the reverse-phase evaporation method.^56^ Briefly, the encapsulant (10 mM SRB and 210 mM NaCl) was dissolved in 4.5 mL of 0.02 M HEPES buffer, pH 7.5. Lipids were dissolved in 3 mL chloroform and 0.5 mL methanol and sonicated for 1 min at 60 °C. A 2 mL encapsulant solution was added to the dissolved lipids and the solution was sonicated for 4 min at 60 °C. Organic solvents were evaporated at a rotary evaporator (LABOROTA 4001, Heidolph, Germany) at 60 °C by stepwise reduction of pressure (900 mbar for 10 min, 850 mbar for 5 min, 800 mbar for 5 min, 780 mbar for 20 min). The solution was vortexed for 1 min, another 2 mL of encapsulant was added, and the solution was vortexed again for 1 min. The residual organic solvents were evaporated at 60 °C (750 mbar for 20 min, 600 mbar for 5 min, 500 mbar for 5 min, 400 mbar for 20 min). The solution was then extruded using polycarbonate membranes with pore sizes of 1, 0.4, and 0.2 μm to obtain unilamellar liposomes. Extrusion was conducted at 65 °C by repeatedly pushing the solution through the syringes (21 repetitions for each pore size). Excess encapsulant was removed by size exclusion chromatography using a Sephadex G-50 column, followed by dialysis against HSS buffer in a dialysis membrane Spectra/Por© 4 (MWCO 12–14 kDa).

### Liposome Characterization

The phospholipid concentration of liposomes was determined by inductively coupled plasma optical emission spectroscopy (ICP-OES) measurements (SPECTROBLUE TI/EOP from SPECTRO Analytical Instruments GmbH, Kleve, Germany). Phosphorus was detected at a wavelength of 177.495 nm. Calibration of the device was performed using phosphorus standard solutions between 0 and 100 μM in 0.5 M HNO_3_. The device was recalibrated before each measurement using 0 and 100 μM standard solutions. Liposome stock solutions were diluted 1:100 or 1:150 in 0.5 M HNO_3_ to determine their total phosphorus content. The total lipid concentration (total lipids) was calculated from the lipid composition used during synthesis.

The hydrodynamic diameter, polydispersity index (PDI) and ζ-potential of liposomes were determined by dynamic and electrophoretic light scattering (DLS, ELS) using a Malvern Zetasizer Nano-ZS (Malvern Panalytical, Germany). Liposome stock solutions were diluted to 50 or 25 μM total lipids in HSS buffer (dispersant refractive index: *n*_D_^20^ = 1.34; dielectric constant: ε = 78.5; viscosity: η = 1.1185 mPa·s). Poly­(methyl methacrylate) (PMMA) semimicro cuvettes (Brand, Germany) were used for size determination with an angle of 173° and backscattering mode after equilibration for 15 s at 25 °C in three measurement runs with 13 single measurements each. ζ-potential measurements were carried out in folded capillary cells (Malvern Panalytical, Germany) after equilibration at 25 °C for 60 s in four measurement runs with each 20 single measurements.

The liposomes were further characterized to determine the maximum fluorescence and liposome stability. Therefore, the fluorescence of lysed (after 15 min incubation with 30 mM OG as detergent) and intact liposomes (1 μM total lipids in HSS) was measured with a BioTek SYNERGY neo2 fluorescence reader (λ_ex_ = 560 nm, λ_em_ = 585 nm, BW = 10 nm, gain 100). The unlysed fluorescence was calculated as the ratio of the fluorescence intensities of intact and lysed liposomes.

### Surface Modification of Liposomes with Streptavidin

Streptavidin was coupled to carboxyl groups present on the liposome surface using EDC/NHS chemistry. The liposome surface was activated using EDC and sulfo-NHS (both 10 mg/mL in 0.05 M MES buffer pH 5.5) for 1 h at room temperature (RT) and 300 rpm. A 1:100:180 ratio of carboxyl groups/EDC/sulfo-NHS was used. The respective amount of streptavidin was added, and the solution was further incubated for 1.5 h at RT and 300 rpm. The streptavidin-modified liposomes were dialyzed overnight against HSS buffer in a Spectra-Por Float-A-Lyzer G2 (1 mL, MWCO: 1000 kDa) to remove excess coupling reagents. The total lipid concentration was again determined by ICP-OES measurement.

### Homogeneous Complement Assay

The homogeneous complement-dependent assay contains at least two conditions, each in triplicates: liposomes in active complement serum (aS) or in inactive complement serum (iaS; negative control). Some studies also included liposomes in liposome complement buffer (LCB; negative control) and a separate positive control (lysed liposomes using the detergent OG). The complement serum was inactivated by the addition of an inactivation complement buffer (iaCB, containing 0.2 M EDTA and 0.5 μM EGTA). LCB, sucrose in LCB (0.2 M per well) and iaCB were added to a black, flat-bottom MTP on ice to prevent complement activation before the start of the measurement. Streptavidin-modified liposomes were incubated with biotinylated polymer conjugates for 2 h at RT and 300 rpm. Antibodies were added and the samples were further incubated for 1 h at RT and 300 rpm before being added to the MTP. Finally, complement-active serum was added to the MTP. The fluorescence was measured in 1.5 min intervals for the first 15 min, followed by 5 min intervals for another 105 min at 37 °C. Fluorescence measurements were performed three consecutive times with a BioTek SYNERGY neo2 fluorescence reader (λ_ex_ = 565 nm, λ_em_ = 585 nm, BW = 8 nm, gain 150). Liposomes were lysed through the addition of 30 mM OG to each well and incubated for 15 min at RT and 300 rpm before the fluorescence intensities were measured again.

### Heterogeneous Binding Assay

BSA-biotin (2 μg/mL in PBS, 100 μL) was immobilized in a high binding MTP overnight at 4 °C. The solution was removed, and the plate was blocked with BSA (1 w/v% in PBS-T, 150 μL) for 1 h at RT and 300 rpm. The MTP was washed twice with PBS-T and three times with HSS (each 150 μL) before addition of streptavidin-liposomes (10 μM total lipids in HSS, 100 μL) and incubation for 3 h at RT and 300 rpm. In the case of the competitive assay for characterization of the biotinylated polymer conjugates, streptavidin-liposomes were mixed with the polymer conjugates prior to MTP addition. The plate was washed three times with HSS (150 μL), and bound liposomes were lysed by addition of 30 mM OG in double-distilled water (100 μL; 15 min incubation at RT and 300 rpm). The fluorescence was measured with a BioTek SYNERGY neo2 fluorescence reader (λ_ex_ = 560 nm and λ_em_ = 585 nm, BW = 10 nm, gain 100 or 150).

### C3a ELISA

Complement component C3a was quantified using a commercial human C3a sandwich ELISA kit from Thermo Fisher (BMS2089TWO). The ELISA was carried out according to the user guide test protocol with 100 μL sample or standard volume. Optical density of the samples was determined at 450 nm. The C3a ELISA was also tested for its specificity toward C3a.

### Data Evaluation

All data are presented as the mean ± standard deviation (SD). Raw data from homogeneous complement assays were processed as follows: The mean (end point) fluorescence intensities of the aS condition and the positive control were both corrected for background by subtracting the fluorescence intensities of the negative control (iaS or aS without trigger entity). The corrected fluorescence intensity of the aS condition is then normalized to the corrected fluorescence intensity of the positive control (lysed liposomes using the detergent OG) and called “corrected lysis”.

$$\mathrm{corrected}\;\mathrm{lysis}=\frac{I\left(\mathrm{aS}\right)-I\left(\mathrm{neg}.\;\mathrm{control}\right)}{I\left(\mathrm{pos}.\;\mathrm{control}\right)-I\left(\mathrm{neg}.\;\mathrm{control}\right)}\times 100\%$$

Data were analyzed statistically using OriginPro 2024 Software. A two-sample independent *t* test was performed when comparing two groups, for instance the active serum sample and the inactive serum sample (negative control). To compare three or more different samples, a one-way analysis of variance (ANOVA) with a post hoc Tukey test was performed. *p*-values ≤ 0.05 were considered statistically significant. * *p* ≤ 0.05, ** *p* ≤ 0.01, *** *p* ≤ 0.001, and ns = not significant.

## Results and Discussion

Liposomes were modified with streptavidin and optimized regarding their serum stability and protein surface coverage. They were employed to study biotinylated complement-mediating trigger entities based on different (bio)­polymeric backbones (BSA, 8-Arm-PEG and dextran). The polymer conjugates fulfill two functions: binding to the liposomes, relying on the well-known streptavidin–biotin interaction, and complement activation, in which case they are also referred to as ‘trigger entities’. Their PEG and biotin moieties were utilized to trigger the complement system through binding of respective antibodies and forming a sandwich complex on the liposome surface, which recruits C1q and initiates the downstream complement cascade (Figure 1). After demonstrating that the polymer conjugates can successfully mediate complement activation, further studies have addressed the question of how the molecular weight and trigger moiety density of the polymer conjugates, as well as the liposome cross-linking and spatial arrangements influence complement activation. Lastly, the long-term storage stability of StAv-liposomes and dextran-based conjugates was investigated, too.

**Figure 1:**
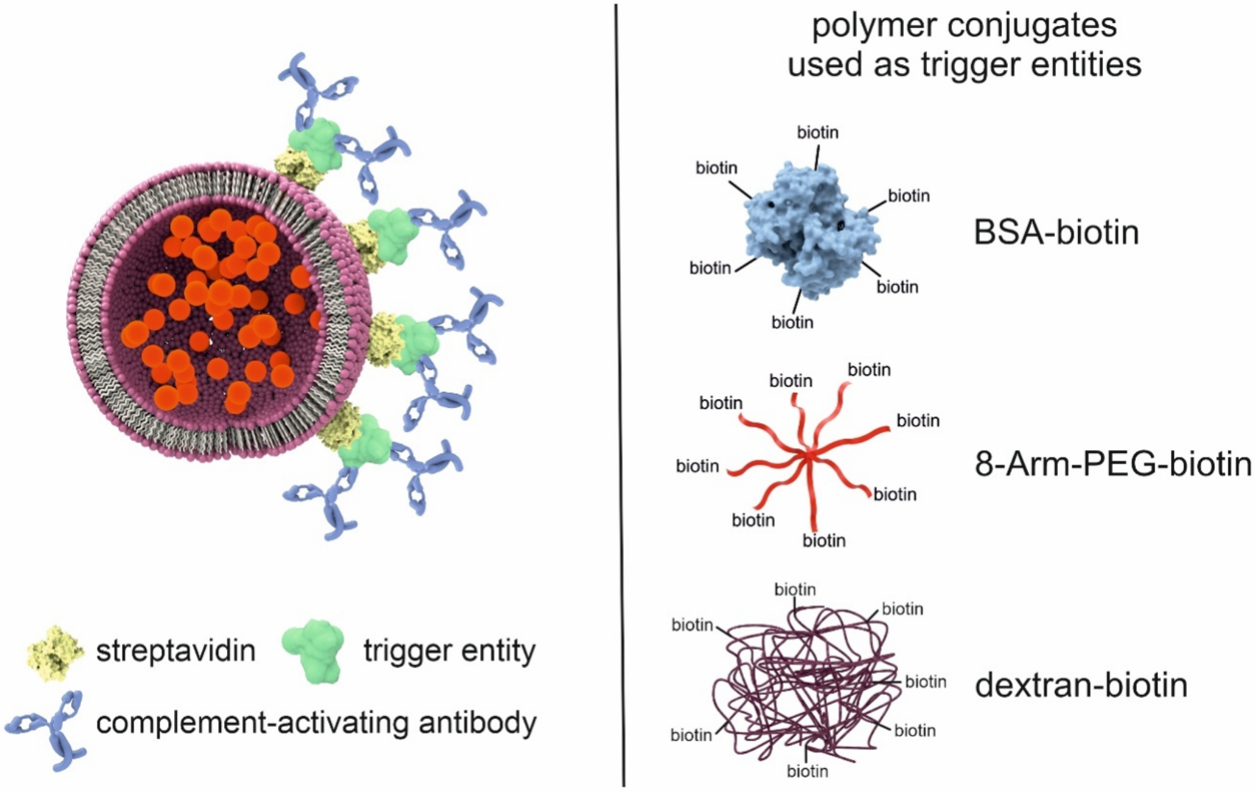
Schematic of the assay principle. Biotinylated polymer conjugates bind to streptavidin-modified liposomes and facilitate binding of multiple complement-activating antibodies, leading to the formation of sandwich complexes on the liposome surface. Activation of the complement cascade leads to the formation of membrane attack complexes, thus liposome lysis and release of the fluorescent dye SRB. The polymer conjugates studied (BSA-biotin, 8-Arm-PEG-biotin and dextran-biotin) are shown on the right.

### Optimization of the Liposome Surface and Streptavidin Loading

Serum stability of liposomes is a key feature to enable their controlled lysis by the complement system. In a previous work, the lipid composition of the liposomes as well as the SRB content were optimized to create stealth liposomes, which are stable in serum. A cholesterol content of 5 mol % and a loading with 10 mM SRB were identified as optimal composition facilitating a sensitive readout and avoiding unintended complement activation, as it is the case for more than 5 mol % cholesterol.^33^ Therefore, using this basic composition, carboxylic acid groups were introduced to the liposomes surface via *N*-glutaryl-DPPE (4 mol %), which were then modified with streptavidin using EDC/sulfo-NHS chemistry. Various streptavidin loadings (0.2–2.5 mol %) of the liposome surface were investigated to optimize the surface coverage. The resulting streptavidin-modified liposomes (StAv-liposomes) were characterized physicochemically using DLS (Table S2) and in a binding assay to immobilized BSA-biotin (Figure 2A) for evaluation of the streptavidin surface loading. In the lower concentration range (0.2–1 mol %), it was found that the more streptavidin was coupled to the liposomes, the better the binding to BSA-biotin due to a higher avidity. In the upper range of 1–2.5 mol %, saturation of the liposome surface with streptavidin appears to occur, as no further improvement in binding of BSA-biotin was observed. It should be noted that the loadings are theoretical values, the actual amount of streptavidin per liposome cannot be determined without substantive effort (such as radionuclide labeling) and would not contribute much to the understanding and development of the StAv-liposomes.

**Figure 2:**
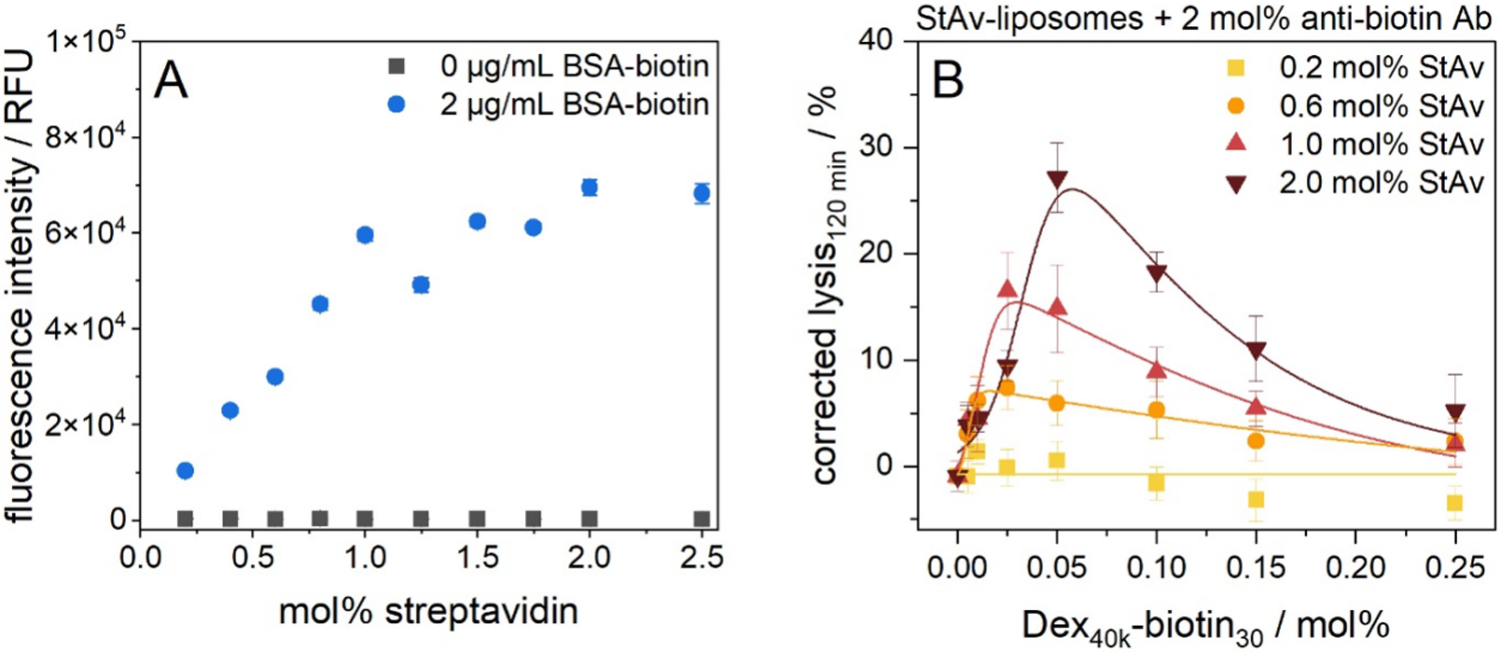
(A) Optimization of the streptavidin loading of the liposome surface using a heterogeneous binding assay. StAv-liposomes StAv1–10 (10 μM total lipids in 100 μL HSS) with varying streptavidin content were immobilized on a high binding MTP coated with 2 μg/mL BSA-biotin. The plate was blocked with 1 w/v% BSA in PBS-T. After washing with PBS-T (2×, 150 μL) and HSS (3×, 150 μL), liposomes were incubated for 3 h at RT and 300 rpm, washed with HSS (3×, 150 μL) and lysed by addition of 30 mM OG in double dist. H_2_O (100 μL, 10 min inc., RT, 300 rpm). λ_ex_ = 560(10) nm and λ_em_ = 585(10) nm, gain 150. *n* = 3. (B) Dependence of the complement-induced liposome lysis on the streptavidin loading. StAv-liposomes StAv1, 3, 5, and 9 (1 μM total lipids) and Dex_40k_-biotin_30_ were incubated for 2 h at RT and 300 rpm. The antibiotin antibody (2 mol %) was added and the samples were further incubated for 1 h at RT and 300 rpm. A 5 vol % human serum was used as complement source (IRS45270). Fluorescence measurements were carried out for 120 min at 37 °C in aS. The liposome samples were lysed after the measurement by addition of 30 mM OG and incubation for 15 min at RT and 300 rpm. Fluorescence intensities were corrected for the negative control (no trigger entity) and normalized to the corrected fluorescence of lysed liposomes. λ_ex_ = 565(8) nm and λ_em_ = 585(8) nm; gain 150. *T* = 37 °C. *n* = 3.

To study the effect of the resulting streptavidin surface coverage on the complement activation, liposomes bearing 0.2, 0.6, 1, and 2 mol % streptavidin were tested in a complement assay using biotinylated dextran (Dex_40k_-biotin_30_) along with an antibiotin antibody as complement trigger. A more detailed description and explanation of the characteristic progression of complement-induced lysis in response to varying polymer conjugate concentrations are provided in the subsequent chapter. Here, we focus initially only on the liposome surface design. It was found that surface loading plays a decisive role in complement activation. While 0.2 mol % StAv-liposomes showed no detectable lysis, complement-induced liposome lysis increased from 0.6 to 2 mol % streptavidin demonstrating that the extent of complement lysis correlates with the amount of streptavidin attached to the liposome surface (Figure 2B). A higher streptavidin loading allows a higher complement trigger density, as more Dex_40k_-biotin_30_ can bind. This increases the probability of C1q binding, which is recruited by the Fc fragments of bound antibodies, facilitating the complement cascade to proceed and ultimately leading to MAC formation.^22^ Furthermore, the higher the streptavidin loading, the higher the polymer conjugate concentration needed for maximum lysis (Figure 2B and Table S3). It can be concluded that the surface coverage of the liposomes and thus the number of binding sites and the trigger density play a key role in complement activation. A surface loading of 2 mol % streptavidin was identified as optimum providing best conditions for polymer conjugate binding and was used for all further studies investigating triggering entities. It should be noted again that the distinct hook-shaped curves (Figure 2B) will be discussed in the subsequent chapter, as they are related to polymer conjugate binding effects.

A common practice in the use of coupling reactions is quenching of unreacted groups to avoid uncontrolled side reactions.^57–59^ To evaluate whether this surface alteration influences complement activation, various quenching agents were tested for their potential to affect complement-mediated lysis (Figure S1 and Table S4). Liposomes quenched with ethanolamine or glycine remained stealth in active serum, whereas the use of PEG-amine, cysteamine, butylamine, or lysine led to substantial lysis. Interestingly, omitting a quencher entirely also resulted in stealth properties. In this case, the activated NHS ester undergoes hydrolysis, regenerating the original carboxyl group on the liposome surface. The hydrolysis half-lives of NHS-esters are typically in the range of 2–5 h at neutral pH, but also depend on temperature and NHS species,^60,61^ suggesting that most NHS esters are hydrolyzed after 24 h of dialysis. As hydrolysis represents the most straightforward and least invasive method, i.e., minimally altering the liposome surface and thereby reducing the risk of undesired complement activation, it was selected for use in subsequent experiments with StAv-liposomes. Another critical parameter for liposome stealthiness is the liposome-to-serum ratio, which must be adapted to the respective serum batch due to the different inherent complement activities, as reported previously.^33^ Therefore, a serum titration was carried out and 5 vol % human serum (batch IRS45270) was determined as optimum concentration at which 2 mol % StAv-liposomes continue to remain stealth, as there was no significant difference in liposome lysis between active and inactivate serum. While the use of more serum would probably yield even higher complement lysis values, the liposomes suffer from an increased background signal under those conditions due to nonspecific complement activation (Figure S2).

### Evaluation of Protein, PEG and Polysaccharide Backbones as Complement Trigger Entities

It is well-known that binding of a complement trigger to liposomes causes their lysis.^33,62–65^ As described above, such a complement trigger can be an antibody that recognizes a specific structure on the liposomal surface. Here, we expand this concept by introducing mediating molecules, i.e., complement triggers will bind to the liposome surface only in the presence of those mediators. Such mediators or trigger entities consist of a (bio)­polymeric backbone structure, a liposome-binding moiety and a complement trigger binding moiety. Since the focus was on the backbone structure, the well-established streptavidin–biotin chemistry was chosen for the liposome-binding mechanism. Similarly, antibiotin and anti-PEG antibodies, which had previously been identified as effective complement triggers, were applied here.^33^ Thus, the polymer conjugates have a dual function which is on the one hand the specific binding to the liposomal surface and on the other hand promoting complement activation (Figure 1). Various molecular structures—including proteins, polymers and polysaccharides—were investigated regarding their suitability for realizing both functions in one entity. In particular, BSA, dextran and an 8-Arm-PEG were studied as biotinylated backbone structures (Figure 1) in the homogeneous complement assay. Here, StAv-liposomes were first incubated with polymer conjugates before addition of the antibodies and subsequent processing steps such as serum addition and measurement (Figure S3).

In a first step, it was necessary to investigate if MAC insertion into the liposomes—and thus complement-induced lysis—remains feasible when using a polymer conjugate as trigger entity, as the trigger molecule is located farther away from the liposomal surface. Therefore, complement-mediated liposome lysis when using a polymer conjugate was compared to regular complement activation, i.e., direct antibody binding to a structure on the liposome surface. This was studied using a time-resolved fluorescence readout (Figures S4 and S5). Exemplary, time-resolved curves of Dex_40k_-biotin_30_-induced and regular complement activation were fitted using a delayed exponential association model to analyze the kinetics of the fluorophore release (Figure S6 and Table S5). The same amount of antibiotin antibody (2 mol %) and a similar total amount of biotin (n_biotin, Dex_ = 1.2 pmol, n_biotin, Liposome_ = 2.0 pmol) were used. The release of SRB and thus complement-induced liposome lysis occurs not only significantly faster (τ = 19 min vs 39 min) when the complement trigger binds directly to the biotinylated liposome surface but also begins earlier (onset after 8 min) compared to the Dex_40k_-biotin_30_-mediated approach (onset after 19 min). This suggests that the recruitment and assembly of complement proteins takes considerably longer using polymer conjugates, likely due to a different distribution of trigger moieties, reduced trigger moiety accessibility and increased complexity of the system compared to direct antibody binding. Even though complement activation varies depending on the properties of the polymer conjugate used, the assay runtime was extended from 60 to 120 min, which had been established previously.^33^ This adjustment significantly enhanced both the sensitivity and dynamic range of the assay.

After confirming the feasibility of using polymer conjugates as trigger entities, the different backbone structures were compared in a concentration-dependent titration with respect to their ability to induce complement activation and to determine the optimum concentration for each polymer conjugate. This titration of the polymer conjugates typically resulted in a sharp increase in complement-induced lysis, followed by an exponential decline (Figures 3A–D and 2B). Initially, the density of complement triggers on the liposomes increases as more polymer conjugates bind until the liposome surface is completely saturated. Beyond this point, excess polymer conjugates remain unbound in solution, capturing antibodies and competing with liposome-bound polymer conjugates, thereby reducing the effective trigger density on the liposomes and resulting in a hook effect. The highest extent of complement lysis (71%) was achieved using the 8-Arm-PEG_10k_-biotin_8_ along with antibiotin and anti-PEG antibodies as triggers (Figure 3A). BSA-biotin_9_ (58%) and Dex_40k_-biotin_30_ (38%) induced lower levels of complement lysis but also contained only one triggering antibody type (Figures 3B,C). The structural features of the 8-Arm-PEG-biotin allowed the use of two different antibodies as complement triggers (Figure S7), thereby increasing the likelihood and efficiency of complement activation. Previously, it was demonstrated that the use of two different antibodies bound to the liposome surface facilitates a synergistic effect that further enhances complement lysis.^33,34^

**Figure 3:**
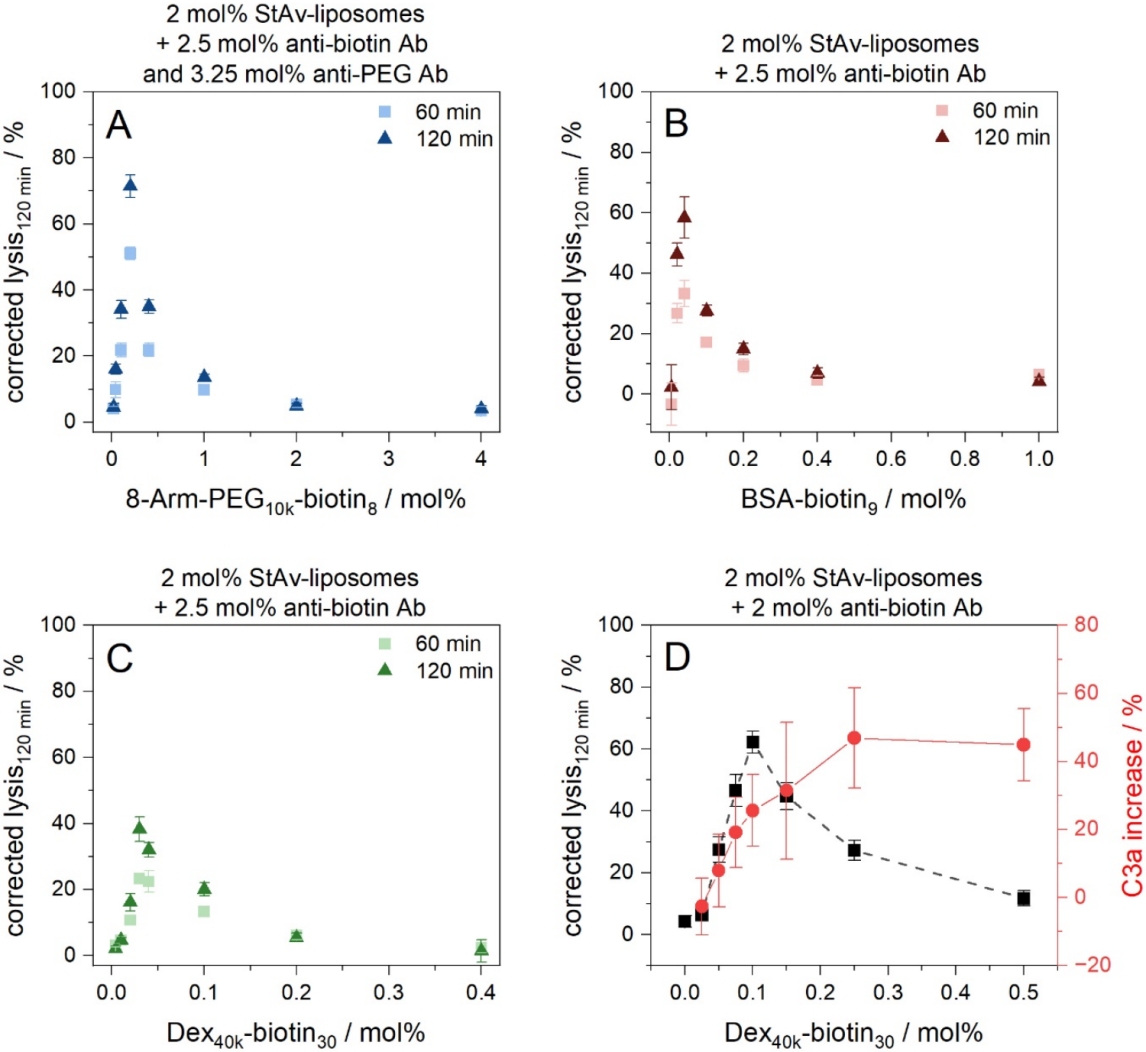
Study of different biotinylated polymer conjugates as trigger entities inducing complement lysis of StAv-liposomes: 8-Arm-PEG_10k_-biotin_8_ (A), BSA-biotin_9_ (B) and Dex_40k_-biotin_30_ (C). StAv-liposomes (StAv11) and varying concentrations of biotinylated polymer conjugates were incubated for 2 h at RT and 300 rpm. Antibodies were added and the samples were further incubated for 1 h at RT and 300 rpm. A 5 vol % human serum was used as complement source (IRS45270). Fluorescence measurements were carried out for 120 min at 37 °C in aS. The liposome samples were lysed after the measurement by addition of 30 mM OG and incubation for 15 min at RT and 300 rpm. The aS samples were corrected for the negative control (iaS) and normalized to the corrected fluorescence of lysed liposomes. λ_ex_ = 565(8) nm and λ_em_ = 585(8) nm; gain 150. *T* = 37 °C. *n* = 3. (D) Correlation of complement-induced StAv-liposome lysis (StAv13) triggered by Dex_40k_-biotin_30_ and C3a generation determined in a C3a ELISA as proof of complement activation.

Overall, the initial results confirm the feasibility of using polymer conjugates as trigger entities for liposome lysis instead of only an antibody. Furthermore, the findings indicate that the extent of complement-induced lysis as well as the optimum polymer conjugate concentrations depend on several factors including the degree of biotinylation, the accessibility, distribution and orientation of the biotin groups, and the overall size of the polymer conjugates. Indeed, the importance of the binding accessibility was confirmed when the influence of antibody quantity on complement lysis was investigated using Dex_40k_-biotin_30_ and 8-Arm-PEG_10k_-biotin_8_ (Figure S8). Overall, it was found, not surprisingly, that higher antibody concentrations lead to more complement-induced lysis. In case of Dex_40k_-biotin_30_, saturating antibody concentrations of 1–1.5 mol % were determined. In contrast, relatively high antibody concentrations (2.5 mol %) were applied in initial experiments to ensure substantial complement activation (Figure S3A–C). In the end, a concentration of 2 mol % antibiotin antibody was chosen for subsequent experiments as a standardized condition. In case of the 8-Arm-PEG_10k_-biotin_8_, different combinations of anti-PEG and antibiotin antibodies were evaluated (Figure S8B). It was found that the antibiotin antibody itself did not lead to any complement triggering. This suggests that the distance between biotin moieties is too large to serve in its desired function. (Figure S8B). Instead, closer spacing created by additional binding of anti-PEG antibodies is required for complement triggering, i.e., C1q binding.

To confirm that the observed liposome lysis is a result of complement activation, an ELISA was conducted following the liposome-based complement assay to quantify the anaphylatoxin C3a, a cleavage product of complement protein C3. The cleavage product C3b contributes to the formation of C5 convertase, a crucial step required for activation of the terminal complement pathway. Therefore, elevated C3a levels strongly suggest progression of the downstream complement cascade. As a representative example, this study was conducted with Dex_40k_-biotin_30_ as trigger entity. Complement activation was found to operate in two phases: Initially, the C3a generation increases and correlates with complement-induced lysis of StAv-liposomes (Figure 3D). At high concentrations of Dex_40k_-biotin_30_, the liposome surfaces become saturated, and any further increase results in the presence of free Dex_40k_-biotin_30_. These unbound polymer conjugates compete with liposome-bound polymer conjugates for binding to the triggering antibodies, leading to the formation of antibody-polymer conjugate complexes in solution. Although C3a generation continues, the antibodies and complement activation are effectively redirected away from the liposome surface, leading to reduced liposome lysis despite ongoing complement activation in solution. This demonstrates that liposomes remain stealth, as complement activation occurs in solution without causing a bystander effect on the liposomes. Saturation of C3a generation, and hence complement activation, occurs at 0.25 mol % Dex_40k_-biotin_30_ due to depletion of complement proteins or, more likely, the triggering antibiotin antibodies. To confirm that complement activation occurs via the classical pathway, a C1q inhibitor was applied to block the interaction between C1q and the complement-triggering antibodies and thus the following complement cascade (Figure S9). In presence of the inhibitor, no lysis of StAv-liposomes was observed, indicating that complement activation was effectively suppressed, and that the classical pathway is responsible for initiating the complement response.

### Enhancing Complement Lysis by Tuning the Molecular Weight and Biotinylation Degree of the Dextran-Based Polymer Conjugates

Next, the molecular weight of the dextran-biotin and the 8-Arm-PEG-biotin as well as the biotinylation degree of the dextran were tuned. ^1^H-NMR measurements were performed to determine the biotinylation degrees of the polymer conjugates (Figures S16–S26). Polysaccharides such as dextran are particularly well suited for such studies, as their polymeric and uniform structure allows a high level of synthetic control. For dextran-biotin, molecular weights of 10 and 40 kDa were examined, while for the 8-Arm-PEG-biotin, 10, 20, and 40 kDa variants were investigated. In addition to the homogeneous complement assay, all polymer conjugates were also evaluated in a competitive heterogeneous binding assay to further assess their biotinylation degree and biotin accessibility. Here, StAv-liposomes were incubated with varying concentrations of the polymer conjugates and allowed to bind to a BSA-biotin-coated MTP (Figure S10). Depending on the extent of liposome surface coverage with polymer conjugates, binding to the immobilized BSA-biotin was either possible or inhibited. BSA-biotin was not included in these studies as it was purchased commercially and generally allows less synthetic control than the other polymer conjugates. In general, its EC_50_ value was comparable to Dex_10k_-biotin_24_ and 8-Arm-PEG_10k_-biotin_8_ (Figure S11D and Table S6) studied below. Hence, the degree of biotinylation was studied using the dextran backbone, as this was able to cover the largest biotinylation range.

Coupling of biotin to the backbone resulted in 5 to 24 biotins per dextran molecule for the 10 kDa dextran, and 24 to 133 biotins per dextran molecule for the 40 kDa dextran. Not surprisingly, the coupling efficiency increased with biotin surplus (Table S6). The degree of substitution (DOS) was calculated to enable the comparison of dextrans with different molecular weights. Highly substituted (DOS > 0.4) dextran-based polymer conjugates were insoluble in water but could be dissolved in DMSO and further diluted in buffers for use. Studying the polymer conjugates in the competitive binding assay revealed the trend toward lower EC_50_ values with increasing biotinylation degree of dextran-biotin conjugates (Figure S11A,B and Table S6). Solely the highest substituted Dex_40k_-biotin conjugate turned out to be an exception, as it was not water-soluble at all and immediately precipitated when diluted in buffer. Furthermore, Dex_40k_-biotin exhibited generally lower EC_50_ values than Dex_10k_-biotin, which can be attributed to the higher average number of biotins per dextran molecule, thus a higher avidity. The 8-Arm-PEG-biotin conjugate revealed lower EC_50_ values for higher molecular weights, which may be attributed to the longer polymer arms enable an enhanced flexibility and accessibility of biotin moieties, which benefits the binding assay (Figure S11C and Table S6). Considering that the higher molecular weight conjugates had fewer biotin moieties and hence should in theory bind less, further supports this conclusion.

Applying these dextran-biotin conjugates as trigger entities in the complement assay along with StAv-liposomes and triggering antibiotin antibodies, they also showed the expected trend, i.e., higher biotinylation degrees promote higher complement trigger densities on the liposome surface and thus enhance liposome lysis (Figures 4A and S12A,B). However, higher biotinylation degrees may also promote liposome cross-linking, which can likewise contribute to enhanced complement activation. The aspect of liposome cross-linking will be discussed in more detail later. Nevertheless, it can hence be confirmed that there is a direct correlation between biotinylation degree and complement lysis, which also matches with the results from the competitive binding assay, showing a correlation of EC_50_ values and complement-induced lysis. Dex_40k_-biotin outperformed Dex_10k_-biotin in this respect, owing to its considerably higher total biotin content per polymer conjugate, even though both exhibit a comparable DOS. Not surprisingly, the only exception is the highest substituted Dex_40k_-biotin due to its complete insolubility in aqueous solutions, leading consequently also to essentially no complement activation. Furthermore, the results indicate that low biotinylation of polymer conjugates is insufficient to efficiently activate the complement system, but that multimers are required, which is consistent with the literature demonstrating that IgG oligomerization promotes efficient C1q recruitment.^42–44^

**Figure 4:**
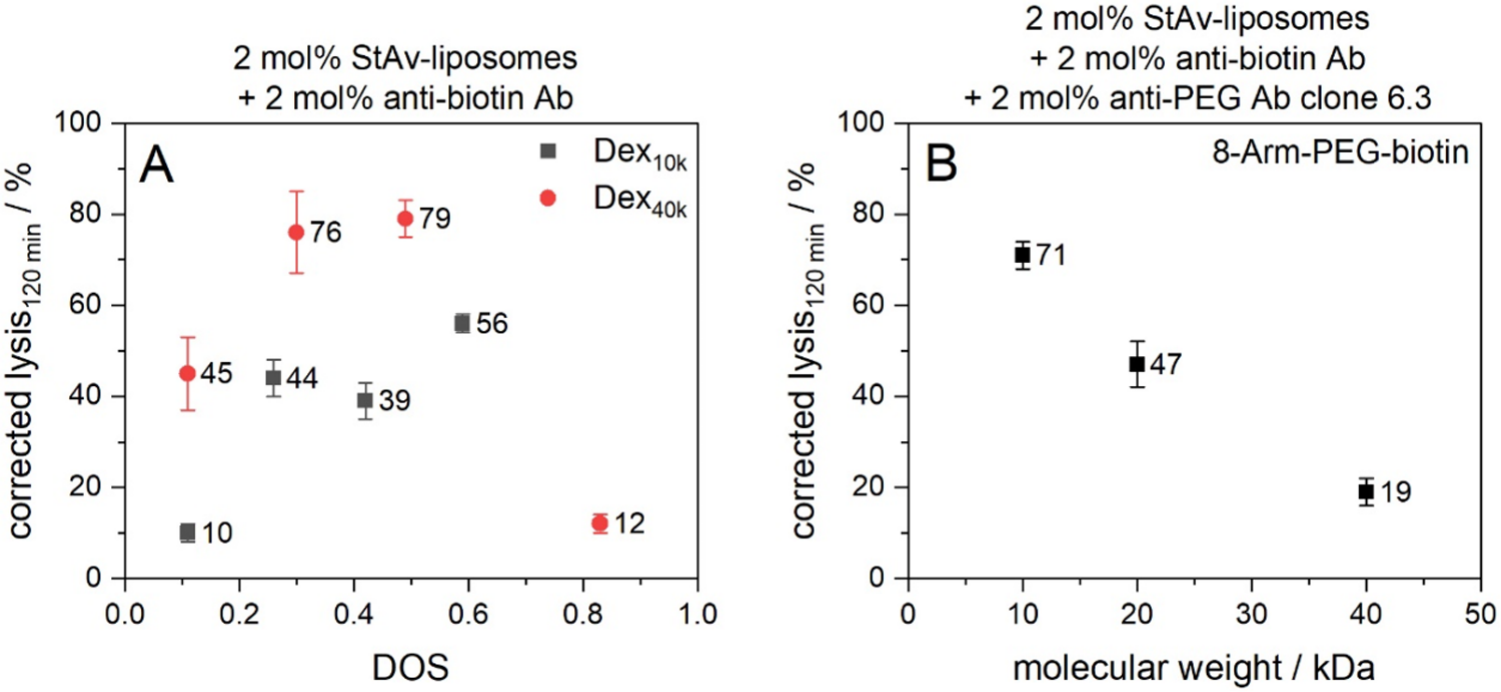
(A) Dependence of complement lysis on DOS of dextran-biotin polymer conjugates. (B) Dependence of complement lysis on molecular weight of 8-Arm-PEG-biotin polymer conjugates. StAv-liposomes (StAv12) and biotinylated polymer conjugates were incubated for 2 h at RT and 300 rpm. Antibodies were added and the samples were further incubated for 1 h at RT and 300 rpm. 5 vol % human serum was used as complement source (IRS45270). Fluorescence measurements were carried out for 120 min at 37 °C in aS or iaS. The liposome samples were lysed after the measurement by addition of 30 mM OG and incubation for 15 min at RT and 300 rpm. The aS samples were corrected for the negative control (iaS) and normalized to the corrected fluorescence of lysed liposomes. λ_ex_ = 565(8) nm and λ_em_ = 585(8) nm; gain 150. *T* = 37 °C. *n* = 3.

A different picture emerged when studying different molecular weights of 8-Arm-PEG-biotin as trigger mediator. Here, higher molecular weights substantially reduced complement-induced lysis of StAv-liposomes (Figures 4B and S12C) albeit having similar biotinylation degrees and suggesting enhanced binding accessibility with higher molecular weight. We therefore assume that the dramatic change of the molecular weight and hence size of the 8-Arm-PEG conjugates is considered as primary factor influencing complement activation in this system. This is consistent with the necessity of antibody proximity for efficient C1q binding, as suggested by structural analyses reporting physiological distances of 8.8–17.9 nm between C1q binding sites on antibodies within C1q-antibody complexes.^25,66^ A closer look at the structural features of the polymer conjugates and their behavior in solution and when bound to the liposome surface provides further insight. Specifically, although dextran, being a nearly unbranched chain,^67^ and 8-Arm-PEG as a star polymer exhibit fundamentally different structures, their behavior in solution is similar. Both will fold into a roughly spherical shape^68^ with comparable hydrodynamic diameters of 4.8 ± 0.2 nm for a 8-Arm-PEG_20k_ and 5.3 ± 0.9 nm for a Dex_40k_-biotin_16_, as determined by DLS (Figure S13). However, complement activation was found to show opposite trends in dextran and 8-Arm-PEG-biotin with increasing molecular weight: it increases in dextran but decreases in 8-Arm-PEG-biotin. This indicates that the underlying structure of the polymer conjugates cannot be ignored, as the behavior in solution is likely to bear little resemblance to the actual structure of the polymer conjugate after it has bound to the liposome surface. The main difference between the polymer conjugates is that 8-Arm-PEG-biotin only has terminal modifications, while dextran is modified along the chain in a random pattern. Consequently, after binding to the liposome surface, PEG retains its range of motion (Figure S14), while dextran most likely attaches at one point along the chain, thereby reducing its range of motion. When multiple points of the dextran chain bind to the liposome, flexibility decreases further, and the polymer might spread across the entire surface (Figure S14). While this explanation seems very plausible, further structural investigations would be required in the future to confirm this hypothesis. The steric flexibility of 8-Arm-PEG-biotin is probably disadvantageous here, as the initiation of the complement cascade occurs too far away from the liposome surface, preventing the complement proteins from anchoring themselves in the membrane. As known in literature, these findings indicate that complement activation occurs within a defined spatial range close to the membrane surface.^25,42,66^ According to structural analyses, the globular heads of C1q, which facilitate binding to antibodies, typically have a physiological distance of 11–14 nm from the membrane surface. When antibodies are located further away, pore formation is reduced or may not occur, as the MAC is unable to effectively insert into the lipid bilayer. Cleary et al. investigated how the epitope distance from the cell membrane influences the complement-dependent cytotoxicity and demonstrated that complement-mediated cell lysis significantly diminishes when the antigen was 16 nm away from the cell surface.^69^ Furthermore, structural analyses have reported distances of 8.8–17.9 nm between C1q binding sites on antibodies, reflecting the spatial requirements owing to the distinct hexameric structure of C1q, often described as a bouquet of flowers.^25,66,70^ Considering the dimensions of the 8-Arm-PEG_40k_, each arm (PEG5000) consists of approximately 114 monomers. Assuming a monomer length of 0.35 nm,^71^ the fully stretched out length of a single arm would be approximately 40 nm. However, polymer chain dimensions are commonly described using Gaussian chain statistics, in which the polymer is modeled as a freely jointed chain of *N* Kuhn segments, each of length *b*.^72^ PEG typically adopts a random coil conformation in aqueous solutions, which in the ideal case can be described by a random walk model. Here, the mean square end-to-end distance is given by

$$\left\langle R^{2}\right\rangle =Nb^{2}$$

For PEG, a Kuhn length of approximately 0.76 nm is commonly reported.^73,74^ Based on this value, each PEG5000 arm corresponds to roughly 54 Kuhn segments. This yields an estimated root-mean-square end-to-end distance of approximately 5.6 nm for a single arm. The actual extension of the polymer chain is expected to lie between the fully stretched length and the random coil formation, but under physiological conditions it will probably be closer to the latter. Importantly, complement activation is not mediated by a single PEG arm but involves multiple arms simultaneously. This implies not only an increased distance between the membrane surface and the trigger moieties, but also a greater spatial separation between individual trigger moieties. Consequently, this supports the hypothesis that the terminal biotin groups of the 8-Arm-PEG—which function both as binding ligand to the liposome surface and as trigger moieties—are positioned too far from the membrane surface or from each other to efficiently initiate pore formation. This also explains why the relationship between EC_50_ obtained from the competitive binding assay and complement lysis is inverse for the 8-Arm-PEG-biotin. Although higher molecular weights enable a higher accessibility of biotin moieties, the accompanying larger spatial extension hampers efficient complement activation.

### The Influence of Liposome Cross-Linking and Different Incubation Strategies on Complement Lysis

Since the use of polymer conjugates with multiple biotin moieties inevitable also leads to cross-linking of StAv-liposomes, the effect of such cross-linking on complement activation was investigated. Therefore, the vesicles size and size distribution were determined in the absence of serum to decouple the phenomena of cross-linking and complement activation. The progression of complement lysis and liposome size followed a similar trend (Figure 5A and Table S7). Low Dex_40k_-biotin_30_ concentrations caused neither complement lysis nor significant cross-linking. Above a critical polymer conjugate concentration, both liposome cross-linking and complement lysis increased abruptly, albeit the onsets were slightly shifted. At higher Dex_40k_-biotin_30_ concentrations, the characteristic hook effect occurred due to excess polymer conjugates, and both liposome size and lysis returned to near baseline values. These findings may indicate a relationship between liposome cross-linking and complement activation, although the two effects could also occur independently of each other. Previous publications have shown that physicochemical characteristics of liposomes, including morphology, size, curvature and aggregation, modulate complement activation and opsonization.^75,76^ Generally, larger liposomes trigger stronger complement responses than smaller ones.^49–51^ Moreover, some studies suggest that liposome aggregation or cross-linking promotes complement activation,^39,52,53^ supporting the theory that liposome cross-linking observed here can likewise enhance complement triggering. Considering the results of the study in which the biotinylation degree of the dextran-based polymer conjugates was tuned, a higher biotinylation degree can also promote liposome cross-linking, which in turn may contribute to enhanced complement activation. An alternative system using separate binding and trigger moieties would be required to decouple both effects.

**Figure 5:**
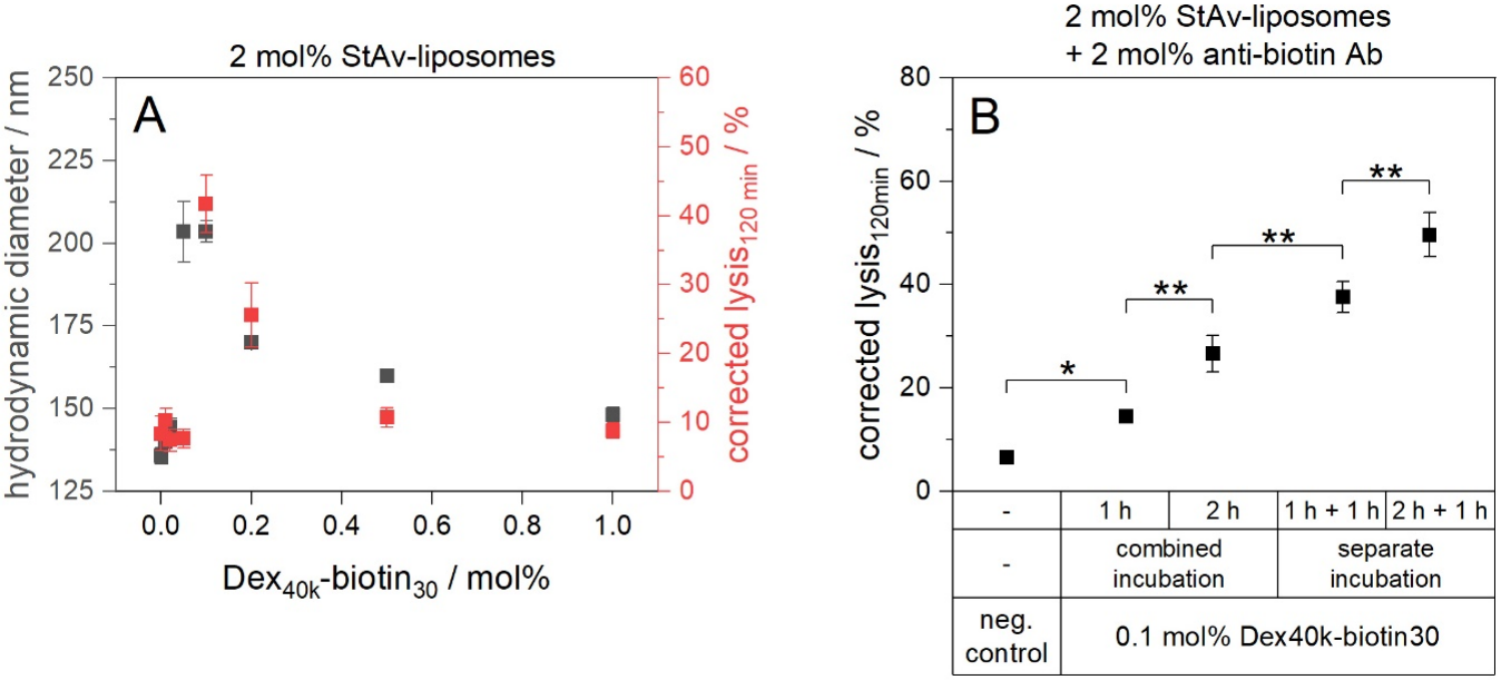
(A) Correlation of complement-induced lysis and StAv-liposome cross-linking determined using DLS in the absence of serum. (B) Study of different incubation strategies. StAv-liposomes (StAv13) and Dex_40k_-biotin_30_ were incubated for 2 h at RT and 300 rpm. Antibiotin antibodies were added and the samples were further incubated for 1 h at RT and 300 rpm. 5 vol % human serum was used as complement source (IRS45270). Fluorescence measurements were carried out for 120 min at 37 °C in aS. The liposome samples were lysed after the measurement by addition of 30 mM OG and incubation for 15 min at RT and 300 rpm. The aS samples were corrected for the negative control (iaS) and normalized to the corrected fluorescence of lysed liposomes. λ_ex_ = 565(8) nm and λ_em_ = 585(8) nm; gain 150. *T* = 37 °C. *n* = 3.

In a further study, different incubation strategies combining StAv-liposomes, Dex_40k_-biotin_30_ and antibiotin antibodies were explored to potentially simplify and streamline the assay workflow. However, it was found that both separate incubation of the polymer conjugate and triggering antibody, as well as prolonged incubation times, were beneficial for complement-induced lysis (Figure 5B). This again reflects the strong dependence of complement activation on spatial arrangement. In the case of a combined incubation, the antibiotin antibody and the StAv-liposomes compete for binding to the most accessible biotin residues on the dextran. As a result, the binding of StAv-liposomes is likely hindered, which is a crucial prerequisite for complement activation, as it allows the assembly of the sandwich complex consisting of streptavidin, polymer conjugate and antibodies required for pore formation. In contrast, during separate incubation, the dextran first associates with the liposomes, followed by antibody binding to the remaining free biotin moieties. This process may promote formation of sandwich complexes and therefore complement activation, as it better mimics the natural geometry of trigger and recognition sites during physiological complement assembly. However, methods such as cryo-EM or FRET need to be applied to identify particular structural differences. In any case, separate incubation of the polymer conjugate and antibody is preferred as it provides an enhanced complement-mediated lysis.

### Storage Stability Study

The storage stability of assay components is a key feature for their use in various applications. Thus, important characteristics of the optimized 2 mol % StAv-modified liposomes such as the colloidal stability, binding ability, serum stability and lipid bilayer integrity and thus SRB leakage were monitored up to 36 weeks at 4 °C. In a previous study, carboxyl-modified 10 mM SRB-liposome were shown to be stable (lipid bilayer integrity and serum stability) for more than 40 months at 4 °C or 10 months at RT.^33^ Surface modification with proteins such as streptavidin may shorten the storage stability, since protein degradation could lead to a reduction in binding ability of the liposomes and thus compromise their overall assay performance. The addition of stabilizing agents such as polysaccharides, polymers, amino acids, proteins or detergents is a common strategy to preserve the functionality of proteins^77^—in our case streptavidin—and thereby prevent agglomeration of liposomes and other nanoparticles. Therefore, similar to a previous study on protein-modified liposomes,^34^ different concentrations of BSA (0.01, 0.05, and 0.25 wt %) were investigated as well as StAv-liposomes in HSS buffer without any additives. Liposomes were stored at 250 μM total lipids. Beyond all conditions, StAv-liposomes remained colloidally stable over 36 weeks, showing only a slight increase in hydrodynamic diameter of up to 8 nm (Figure 6B). Solely liposomes with 0.25 wt % BSA deviated, as they exhibited a reduced size (approximately 10 nm smaller) and higher polydispersity (PDI > 0.2) compared to other StAv-liposomes. This is likely an artifact and due to the high concentration of small BSA molecules, which affects the overall particle size distribution in the DLS measurements. In a binding assay, 2 mol % StAv-liposomes were found to maintain their binding ability for up to 36 weeks when BSA is added (Figure 6A). However, the addition of BSA had only a minor supportive effect, as StAv-liposomes without BSA also maintained 85% of their binding ability demonstrating the remarkable stability of streptavidin against degradation, which has already been shown in a previous study investigating StAv-liposomes with a different lipid and encapsulant composition.^59^ Furthermore, StAv-liposomes showed the general trend of decreasing signals and thus liposome binding the more BSA is present, indicating that BSA impairs liposome binding to immobilized BSA-biotin. Depending on the desired application, this effect must be considered. Measurements of the unlysed fluorescence revealed that no SRB leakage occurred and thus the integrity of the lipid bilayer was maintained during the entire storage period regardless of the amount of BSA added (Figure 6C). Serum stability of the StAv-liposomes was confirmed for up to 12 weeks (Figure 6D). After 24 and 36 weeks, a slightly increase in lysis was observed, indicating that aging effects—such as the subtle alteration of the protein structure and the onset of agglomeration—may begin to compromise the serum stability of the liposomes. Nevertheless, with less than 15% lysis, the StAv-liposomes maintained acceptable stealth properties after 36 weeks of storage, which is sufficient for most applications. In conclusion, the exceptional storage stability of StAv-liposomes supports their potential for a wide range of applications.

**Figure 6:**
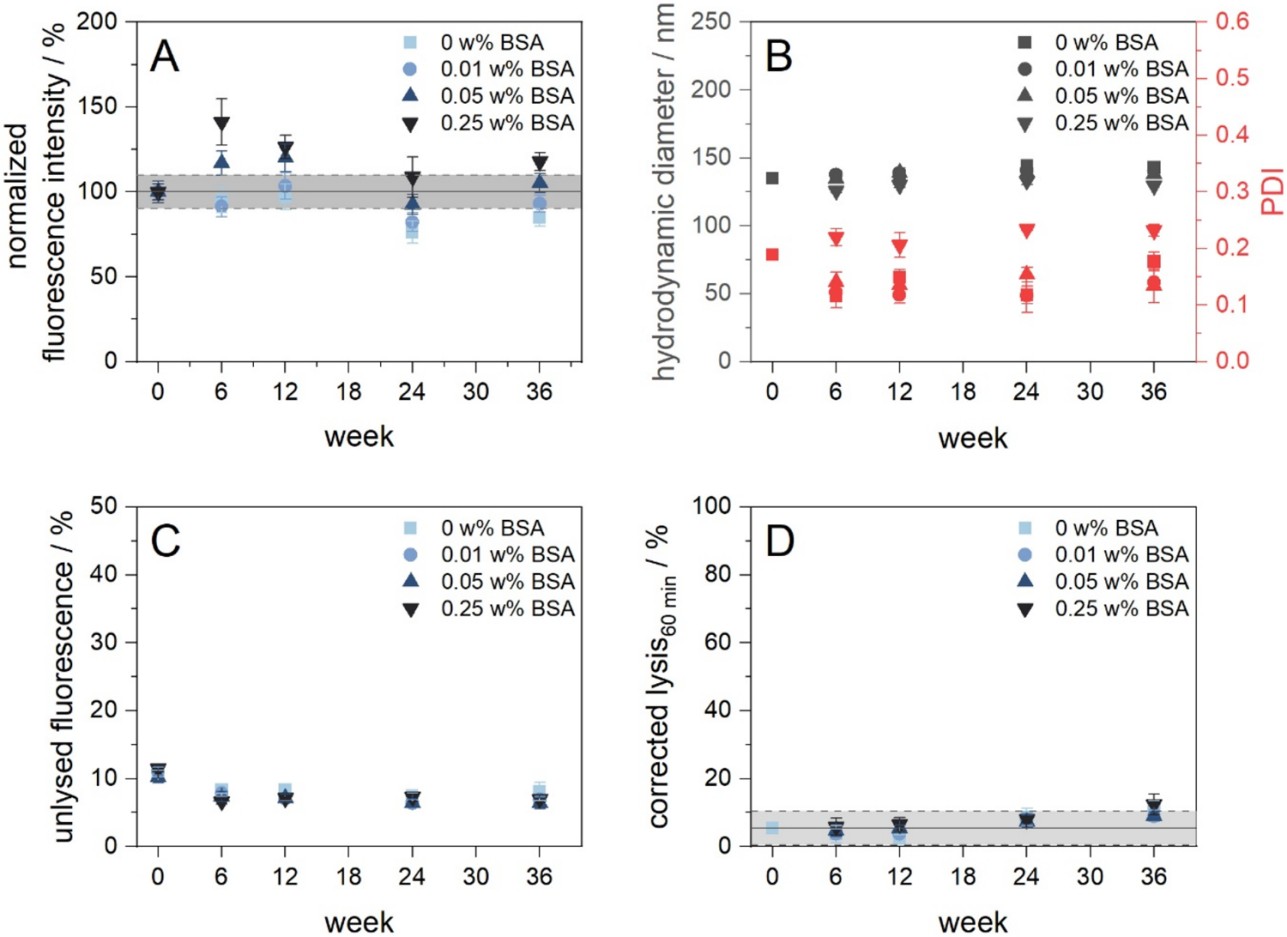
Storage stability study of 2 mol % StAv-modified liposomes (StAv12) monitoring colloidal stability, binding ability and liposome integrity and serum stability. (A) Binding ability of StAv-liposomes to immobilized BSA-biotin in a heterogeneous assay. Liposomes (10 μM total lipids in 100 μL HSS) with varying streptavidin content were immobilized on a high binding MTP coated with 2 μg/mL BSA-biotin. The plate was blocked with 1 w/v% BSA in PBS-T. After washing with PBS-T (2×, 150 μL) and HSS (3×, 150 μL), liposomes were incubated for 3 h at RT and 300 rpm, washed with HSS (3×, 150 μL) and lysed by addition of 30 mM OG in double dist. H_2_O (100 μL, 10 min inc., RT, 300 rpm). λ_ex_ = 560(10) nm and λ_em_ = 585(10) nm, gain 150. *n* = 3. (B) Hydrodynamic diameter and PDI of StAv-liposomes (25 μM total lipids) (C) Unlysed fluorescence of StAv-liposomes (1 μM total lipids) monitoring the lipid bilayer integrity and SRB leakage. λ_ex_ = 560(10) nm and λ_em_ = 585(10) nm, gain 100. *n* = 3. (D) Serum stability of StAv-liposomes (1 μM total lipids) in 5 vol % human serum (IRS45270). λ_ex_ = 565(8) nm and λ_em_ = 585(8) nm; gain 150. *T* = 37 °C. *n* = 3.

The biotin-containing polymer conjugates were also partly tested for their storage stability, as the dextran-based conjugates are linked by ester bounds that may be susceptible to hydrolysis. DOSY-NMR experiments were performed to verify that the dextran-biotin remains functional throughout the duration of the studies. Therefore, a freshly synthesized dextran with 26 biotin residues was subjected to an isothermal stress test at 45 °C for 39 h, after which no visible changes in the spectrum (Figure S15) were observed. We can thus assume that our polymer conjugates are resistant to hydrolysis in aqueous solutions, with lyophilization probably being preferred for long-term storage, which would be part of a future study.

## Conclusions

The complement system is not only a key component of our innate immune system, but also plays an essential role in various diseases, such as systemic lupus erythematosus, age-related macular degeneration, atypical hemolytic uremic syndrome, and even cancer.^27,28^ Despite continuous progress, its mechanisms remain only partially understood, making it an important target for clinical and pharmacological research. Liposomes and lipid nanoparticles are extensively applied in drug delivery, making their design toward a prolonged blood circulation time and reduced clearance of great interest. Furthermore, liposomes can serve as biomimetic models that mimic cellular, bacterial or viral membranes. Hence, a fundamental understanding of their structural and surface features that promote or inhibit complement activation is indispensable. Here, we present a platform technology that enables in-depth studies of the complement system using StAv-modified liposomes and complement trigger-mediating polymer conjugates as model system. Biotinylated trigger entities were composed of PEG (8-Arm), protein (BSA) or polysaccharide (dextran) backbones, and were shown to successfully mediate complement lysis through biotin or PEG moieties that recruit complement-activating antibodies. A C3a ELISA as well as a complement assay under classical pathway conditions were conducted confirming that liposome lysis was mediated by complement activation. Thus, all three polymer backbone chemistries were determined to be feasible structures. When examining different biotinylation degrees of dextran-biotin conjugates, higher biotinylation degrees generally facilitated increased complement-mediated lysis, likely by promoting higher complement trigger densities on the liposome surface or enhancing liposome cross-linking. Also, higher molecular weight backbones increased performance. However, a higher molecular weight of the 8-Arm-PEG-biotin reduced complement lysis as the biotin moieties on the elongated PEG arms might render the trigger antibody to be beyond the physiological distance from the membrane surface for recruitment and anchoring of complement proteins, which is a hypothesis requiring further investigation. Furthermore, investigations of the inevitable cross-linking of StAv-liposomes via polymer conjugates containing multiple biotin moieties revealed a correlation between cross-linking and complement activation, consistent with previous reports that larger surface areas and liposome aggregation enhance complement activation.^49–53^ Lastly, findings suggest that complement activation strongly relies on the spatial arrangement of polymer conjugates and trigger antibody, with processes promoting the formation of a sandwich-like complex on the liposome surface being more favorable for complement activation. These initial findings confirm that this new assay platform provides a deeper insight into liposome surface chemistry, polymer conjugate design and the underlying mechanisms of complement activation. While offering a similar sensitivity and dynamic range as established complement-dependent liposome lysis systems, it allows a higher flexibility and better control of spatial ligand arrangements. It thereby enables the rational tuning of their features for specific applications. Beyond the use of the biotin–streptavidin interaction as a simplified model, the findings demonstrate that the liposome-based complement assay also allows sandwich immunoassays in a no-wash, homogeneous fashion offering an alternative to conventional ELISAs significantly expanding prior studies.^33,34^ Specifically, this new platform technology is highly versatile and can be employed for a wide range of applications, including immunoassays, targeted release of liposome encapsulants for drug delivery systems, and gaining deeper insights into the mechanisms of complement activation and regulation.

## Supplementary Material



## Notes

Detailed information about further chemicals and consumables, buffer compositions, liposome characteristics, quencher study, serum stability of liposomes, antibody titration, pathway-specific lysis, characteristics of polymer conjugates, competitive binding assays of polymer conjugates, titration of polymer conjugates to study the tuning of their biotinylation degree, cross-linking study, flowchart of the homogeneous assay procedure, time-resolved fluorescence curves, fit data, schematics showing assay procedures and hypothesized liposome–polymer conjugate interactions, and NMR spectra of polymer conjugates (PDF)
