# Supplementary material for: Engineering Liposomes and Polymer Conjugates: A Platform for Mechanistic Complement Activation Studies and Controlled Release Applications
Source: ACS Appl Mater Interfaces. 2026 Apr 21;18(17):24178–92. doi: 10.1021/acsami.6c02923 (PMC13154114; doi:10.1021/acsami.6c02923)
Supplement: Supplementary file 1 [file am6c02923_si_001.pdf]

SUPPORTING INFORMATION

Engineering Liposomes and Polymer Conjugates: A Platform for Mechanistic Complement Activation Studies and Controlled Release Applications

Kilian Hoecherl[1‡], Johannes Konrad[2‡], Christina Reiner[1], Simon Streif[1], Clemens Spitzenberg[1], Carola Sommer[3], Diana Pauly[3], Miriam Breunig[2], Antje J. Baeumner[1*]*

[1] Institute of Analytical Chemistry, Chemo- and Biosensors, University of Regensburg, Universitätsstraße 31, 93053 Regensburg, Germany

[2] Department of Pharmaceutical Technology, University of Regensburg, Universitätsstraße 31, 93053 Regensburg, Germany

[3] Experimental Ophthalmology, University of Marburg, Baldingerstraße, 35043 Marburg, Germany

corresponding authors:

*antje.baeumner@ur.de

*miriam.breunig@ur.de

Experimental Section

Chemicals and Consumables

Sucrose, sodium azide, sodium chloride, calcium chloride dihydrate and dialysis membrane Spectra/Por© 4 (MWCO: 12-14 kDa) (2718.1) were purchased from Carl Roth (Karlsruhe, Germany). Phosphorous standard was obtained from Bernd Kraft GmbH (Duisburg, Germany). Chloroform and methanol were purchased from Thermo Fisher Scientific (Waltham, MA, USA). Tetrasodium ethylenediaminetetraacetic acid (EDTA), ethylene glycol-bis (β-aminoethyl ether)-N,N,N',N'-tetraacetic acid (EGTA), disodium hydrogen phosphate dihydrate, potassium dihydrogen phosphate, potassium chloride, magnesium chloride hexahydrate, Whatman Nucleopore™ Track-Etched membranes (1.0 µm, 0.4 µm and 0.2 µm diameter) and Tween® 20 were obtained from Sigma Aldrich/Merck (Darmstadt, Germany).

Buffer Compositions

Table S1: Buffer compositions

**Buffer**	**Composition**
HEPES-saline-sucrose buffer (HSS) pH 7.5	10 mM HEPES
	200 mM NaCl
	200 mM sucrose
	0.01 w/v% NaN_3
Phosphate-buffered saline (PBS) pH 7.4	137 mM NaCl
	2.7 mM KCl
	10 mM Na_2HPO_4
	1.8 mM KH_2PO_4
PBS-T	0.1 w% Tween 20 in PBS
Liposome complement buffer (LCB) pH 7.4	10 mM HEPES
	150 mM NaCl
	135 nM $CaCl_2$
	1 mM $MgCl_2$
MES buffer pH 5.5	0.05 M MES
Inactivation complement buffer (iaCB) pH~8	200 mM EDTA
	0.5 µM EGTA in LCB

Results

Table S2: Characteristics of SRB-encapsulating liposomes. Liposome batch 3 was already used in an earlier publication as batch S12 [1].

batch	Lipid composition	Surface	Encapsulant	Hydrodynamic diameter / nm	PDI	ζ-potential / mV	Total lipid concentration/ mM	Unlysed fluorescence / %
**1**	DPPC: 73 mol%<br>DPPG: 18 mol%<br>Cholesterol: 5 mol%<br>N-Glutaryl-DPPE: 4 mol%	anionic<br>COOH	10 mM SRB<br>210 mM NaCl	114 ± 5	0.10 ± 0.02	-22.4 ± 1.7	9.58 ± 0.10	11.1 ± 1.2
**2**	DPPC: 73 mol%<br>DPPG: 18 mol%<br>Cholesterol: 5 mol%<br>N-Glutaryl-DPPE: 4 mol%	anionic<br>COOH	10 mM SRB<br>210 mM NaCl	138 ± 4	0.10 ± 0.01	-21.8 ± 1.2	10.49 ± 0.02	9.2 ± 0.7
**3**	DPPC: 73 mol%<br>DPPG: 20 mol%<br>Cholesterol: 5 mol%<br>DPPE-biotin: 2 mol%	anionic<br>biotin	10 mM SRB<br>210 mM NaCl	104 ± 2	0.14 ± 0.01	-12.1 ± 0.4	3.42 ± 0.01	9.9 ± 0.8
**StAv1**	batch 1	0.2 mol% StAv	batch 1	113 ± 2	0.17 ± 0.01	-19.4 ± 1.8	0.46 ± 0.01	10.9 ± 1.4
**StAv2**	batch 1	0.4 mol% StAv	batch 1	114 ± 3	0.18 ± 0.02	-21.0 ± 1.6	0.61 ± 0.01	11.0 ± 1.1
**StAv3**	batch 1	0.6 mol% StAv	batch 1	113 ± 1	0.16 ± 0.02	-19.2 ± 3.3	0.58 ± 0.01	10.3 ± 0.8
**StAv4**	batch 1	0.8 mol% StAv	batch 1	114 ± 2	0.19 ± 0.02	-21.1 ± 1.6	0.49 ± 0.01	10.4 ± 0.7

**StAv5**	batch 1	1.0 mol% StAv	batch 1	112 ± 2	0.18 ± 0.01	-21.5 ± 2.3	0.47 ±0.01	9.3 ± 0.5
**StAv6**	batch 1	1.25 mol% StAv	batch 1	112 ± 1	0.19 ± 0.01	-19.1 ± 1.3	0.47 ± 0.01	9.8 ± 0.7
**StAv7**	batch 1	1.5 mol% StAv	batch 1	112 ± 2	0.17 ± 0.01	-19.2 ± 1.6	0.14 ± 0-01	10.0 ± 0.7
**StAv8**	batch 1	1.75 mol% StAv	batch 1	117 ± 3	0.18 ± 0.01	-14.6 ± 2.3	0.44 ± 0.01	8.8 ± 0.8
**StAv9**	batch 1	2.0 mol% StAv	batch 1	118 ± 4	0.21 ± 0.01	-19.4 ± 1.2	0.43 ± 0.01	9.0 ± 0.5
**StAv10**	batch 1	2.5 mol% StAv	batch 1	118 ± 3	0.21 ± 0.01	-18.4 ± 1.6	0.36 ± 0.01	9.2 ± 0.5
**StAv11**	batch 2	2.0 mol% Stav	batch 2	133 ± 2	0.15 ± 0.02	-20.3 ± 1.6	1.19 ± 0.01	11.3 ± 0.6
**StAv12**	batch 2	2.0 mol% Stav	batch 2	135 ± 2	0.19 ± 0.01	-18.8 ± 2.3	1.25 ± 0.01	10.7 ± 0.5
**StAv13**	batch 2	2.0 mol% Stav	batch 2	137 ± 3	0.17 ± 0.03	-16.9 ± 1.2	0.70 ± 0.01	16.3 ± 1.3
**StAv14**	batch 2	2.0 mol% Stav	batch 2	138 ± 1	0.15 ± 0.02	-17.0 ± 1.9	1.92 ± 0.01	12.9 ± 1.3

Table S3: Dependence of the maximum complement-induced liposome lysis on the streptavidin loading. Data were fitted using an asymmetric double sigmoid function in Origin (Asym2Sig). StAv-liposomes (StAv1, 3, 5 and 9) (1 µM total lipids) and Dex_{40k}-$biotin_{30}$ were incubated for 2 h at RT and 300 rpm. Anti-biotin antibody (2 mol%) was added and the samples were further incubated for 1 h at RT and 300 rpm. 5 vol% human serum was used as complement source (IRS45270). Fluorescence measurements were carried out for 120 min at 37 °C in aS. The aS samples were lysed after the measurement by addition of 30 mM OG and incubation for 15 min at RT and 300 rpm. Fluorescence intensities were corrected for the negative control (no trigger entity) and normalized to the corrected fluorescence of lysed liposomes. λ_{ex} = 565(8) nm and λ_{em} = 585(8) nm; gain 150. T = 37 °C. n = 3.

**Streptavidin content**	**Max. corrected lysis**	**Dex_{40k}-$biotin_{30}$**
0.2 mol%	-	-
0.6 mol%	7.1%	0.016 mol%
1.0 mol%	15.4%	0.030 mol%
2.0 mol%	25.3%	0.050 mol%

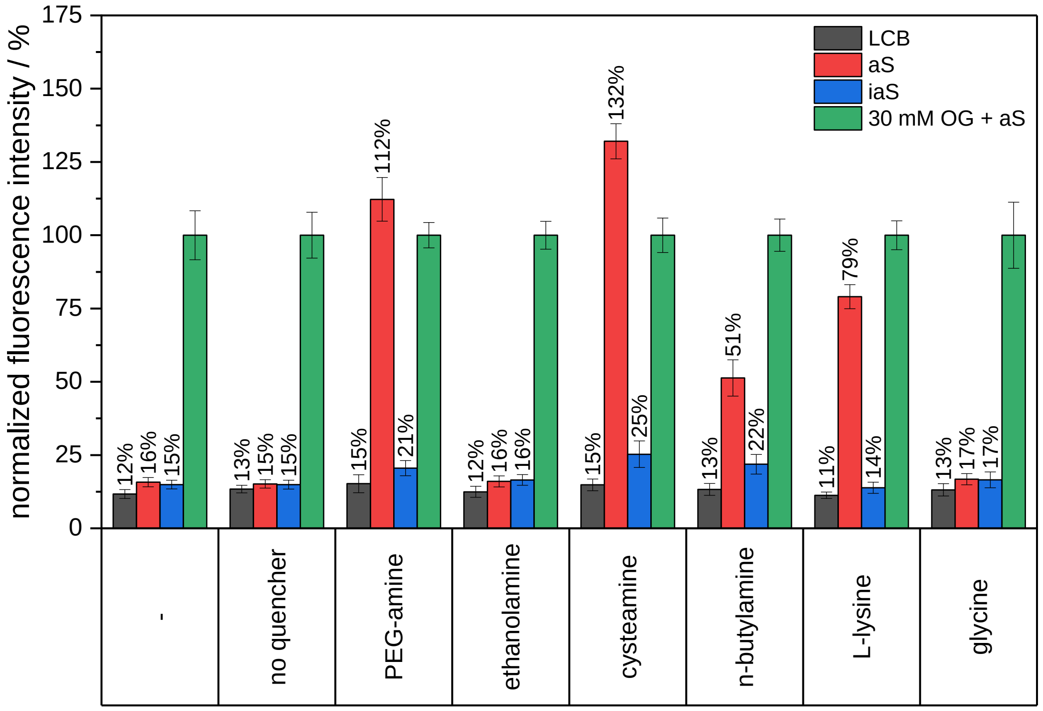

Figure S1: Study of various quencher molecules to ensure serum stability of protein-coupled liposomes (Tab. S4)**.** Carboxyl-liposomes that have not undergone the coupling reaction (-) and liposomes that have been activated with EDC/sulfo-NHS, quenched with varying small molecules containing an amine group or hydrolyzed over time (no addition of any quencher) were tested for their serum stability in a homogeneous complement assay (1 µM total lipids). 10 vol% human serum was used as complement source (IRS38811). Fluorescence measurements were carried out for 1 h at 37 °C in LCB, iaS, aS and 30 mM OG + aS and normalized to the endpoint fluorescence of the positive control. λ_{ex} = 565(8) nm and λ_{em} = 585(8) nm; gain 150. T = 37 °C. n = 3.

Table S4: Characteristics of SRB-encapsulating liposomes (10 mM SRB and 210 mM NaCl as encapsulant) used for the quencher study.

Lipid composition	Surface	Hydrodynamic diameter / nm	PDI	ζ-potential / mV	Total lipid concentra-tion/ mM	Unlysed fluo-rescence / %
DPPC: 73 mol% DPPG: 18 mol% Cholesterol: 5 mol% N-Glutaryl-DPPE: 4 mol%	-	124 ± 2	0.11 ± 0.02	-14.8 ± 2.2	15.87 ± 0.04	3.3 ± 0.9
	no quencher	-	-	-	0.423 ± 0.06	-
	PEG-amine	-	-	-	0.408 ± 0.06	-
	ethanolamine	-	-	-	0.414 ± 0.03	-
	cysteamine	-	-	-	0.407 ± 0.02	-
	n-butylamine	-	-	-	0.419 ± 0.04	-
	L-lysine	-	-	-	0.422 ± 0.09	-
	glycine	-	-	-	0.425 ± 0.04	-

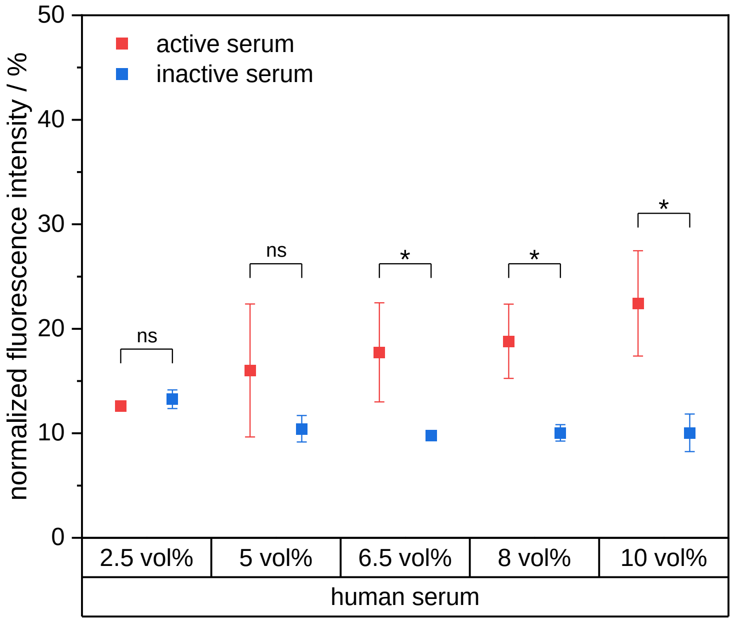

Figure S2: Study of the serum stability of StAv-liposomes (StAv11) in a homogeneous complement assay in 2.5-10 vol% human serum (IRS45270). Fluorescence measurements were carried out for 60 min at 37 °C in aS, iaS (negative control) or 30 mM OG + aS (positive control). Fluorescence intensities were corrected for the negative control (iaS) and normalized to the corrected fluorescence of the positive control. $\lambda_{ex} = 565(8)$ nm and $\lambda_{em} = 585(8)$ nm; gain 150. T = 37 °C. n = 3.

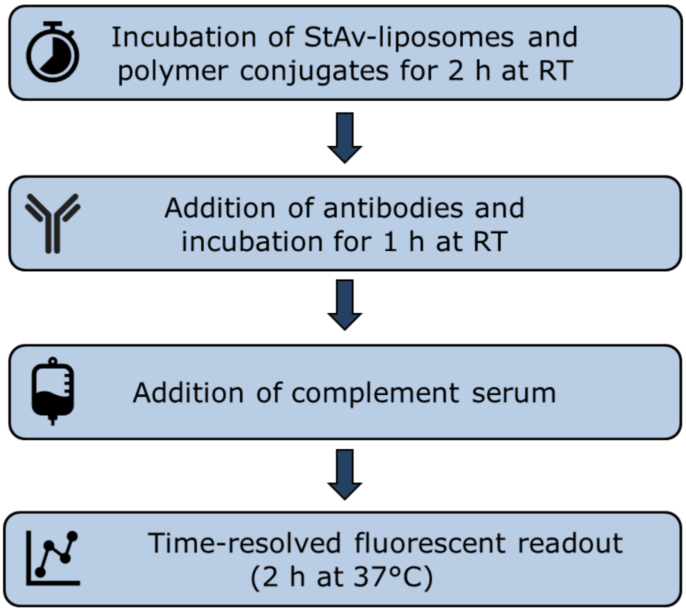

Figure S3: Flow chart of the homogeneous complement assay procedure of StAv-liposomes, polymer conjugates and complement-activating antibodies.

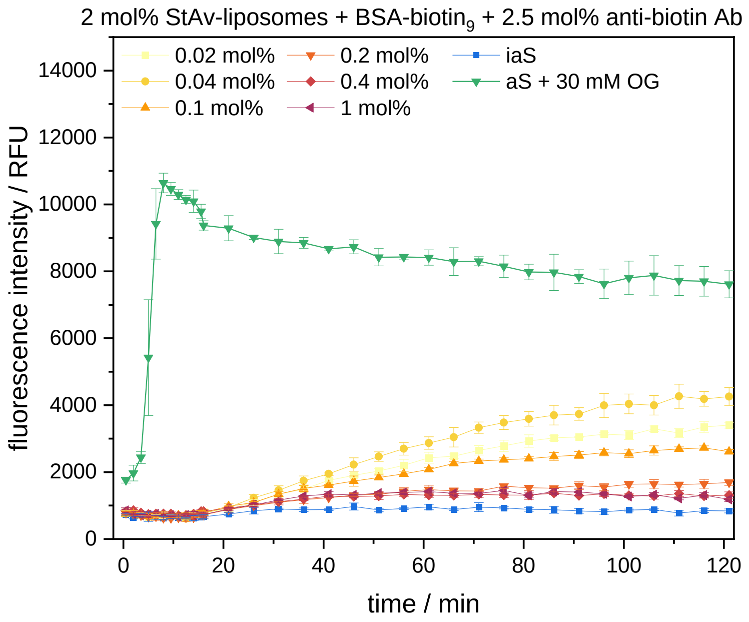

Figure S4: Time-resolved fluorescence intensities of StAv-liposomes (StAv11) in a homogeneous complement assay studying BSA-biotin$_9$ as trigger entity in a titration. StAv-liposomes and varying concentrations of BSA-biotin$_9$ were incubated for 2 h at RT and 300 rpm. Anti-biotin antibodies were added and the samples were further incubated for 1 h at RT and 300 rpm. 5 vol% human serum was used as complement source (IRS45270). Fluorescence measurements were carried out for 120 min at 37 °C in aS, iaS (negative control) or 30 mM OG + aS (positive control). λ_{ex} = 565(8) nm and λ_{em} = 585(8) nm; gain 150. T = 37 °C. n = 3.

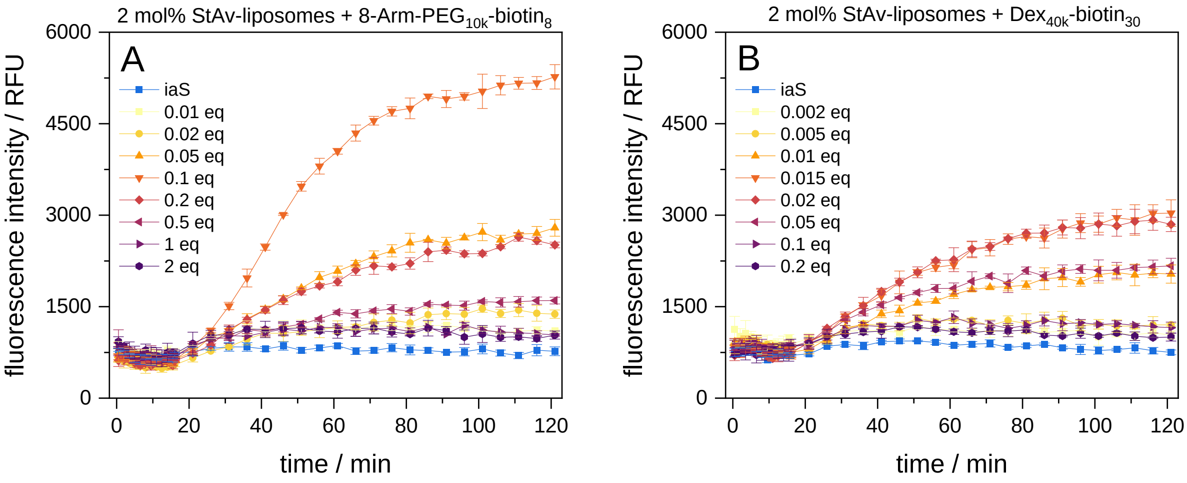

Figure S5: Time-resolved fluorescence intensities of StAv-liposomes (StAv11) in a homogeneous complement assay studying **(A)** 8-Arm-PEG$_{10k}$-biotin$_8$ and **(B)** Dex$_{40k}$-biotin$_{30}$ as trigger entities in a titration. StAv-liposomes and varying concentrations of polymer conjugates were incubated for 2 h at RT and 300 rpm. Anti-biotin antibodies were added and the samples were further incubated for 1 h at RT and 300 rpm. 5 vol% human serum was used as complement source (IRS45270). Fluorescence measurements were carried out for 120 min at 37 °C in aS or iaS (negative control). $\lambda_{ex} = 565(8)$ nm and $\lambda_{em} = 585(8)$ nm; gain 150. T = 37 °C. n = 3.

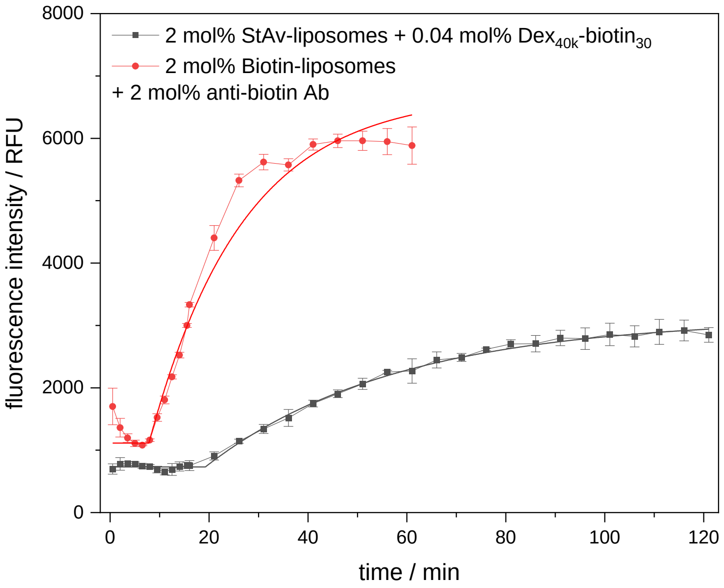

Figure S6: Time-resolved fluorescence intensities of complement-triggered StAv-liposomes (StAv11) or Biotin-liposomes (batch 3) in a homogeneous complement assay. StAv-liposomes and 0.04 mol% Dex_{40k}-$biotin_{30}$ were incubated for 2 h at RT and 300 rpm. Anti-biotin antibodies (2 mol%) were added to StAv- and Biotin-liposomes and the samples were further incubated for 1 h at RT and 300 rpm. 5 vol% human serum was used as complement source (IRS45270). Fluorescence measurements were carried out for 60 or 120 min at 37 °C in aS. Curves were fitted using a delayed exponential association model in Origin. λ_{ex} = 565(8) nm and λ_{em} = 585(8) nm; gain 150. T = 37 °C. n = 3.

Table S5: Fit parameters of the time-resolved complement-induced release of SRB from 2 mol% StAv- or Biotin-liposomes in Fig. S5. StAv-liposomes (StAv11) and 0.04 mol% Dex_{40k}-$biotin_{30}$ were incubated for 2 h at RT and 300 rpm. Anti-biotin antibodies (2 mol%) were added to StAv- and Biotin-liposomes and the samples were further incubated for 1 h at RT and 300 rpm. 5 vol% human serum was used as complement source (IRS45270). Time-resolved curves from Figure S5 were fitted using a delayed exponential association model in Origin.

**Fit parameter**	**StAv-liposomes + Dex_{40k}-$biotin_{30}$**	**Biotin-liposomes**
Time delay TD	19.2	7.8
Baseline value Yb	730	1114
Amplitude A	2381	5597
Time constant τ	38.6	18.9

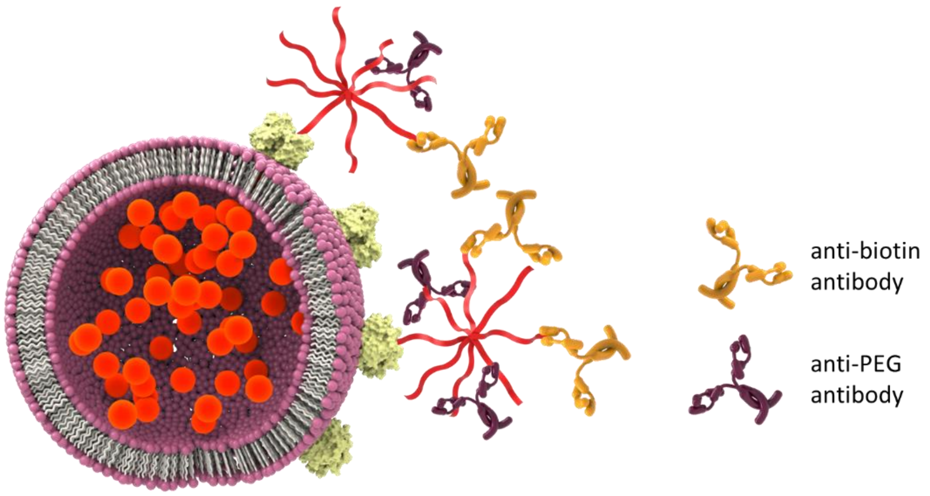

Figure S7: Schematic of antibodies bound to 8-Arm-PEG-biotin on the liposome surface. The structural features of the 8-Arm-PEG-biotin allow the use of two different antibodies as complement trigger and thereby leading to a more efficient complement activation. The anti-biotin antibody targets the terminal biotin moieties of the 8-Arm-PEG-biotin, whereas an anti-PEG antibody targets the PEG backbone structure.

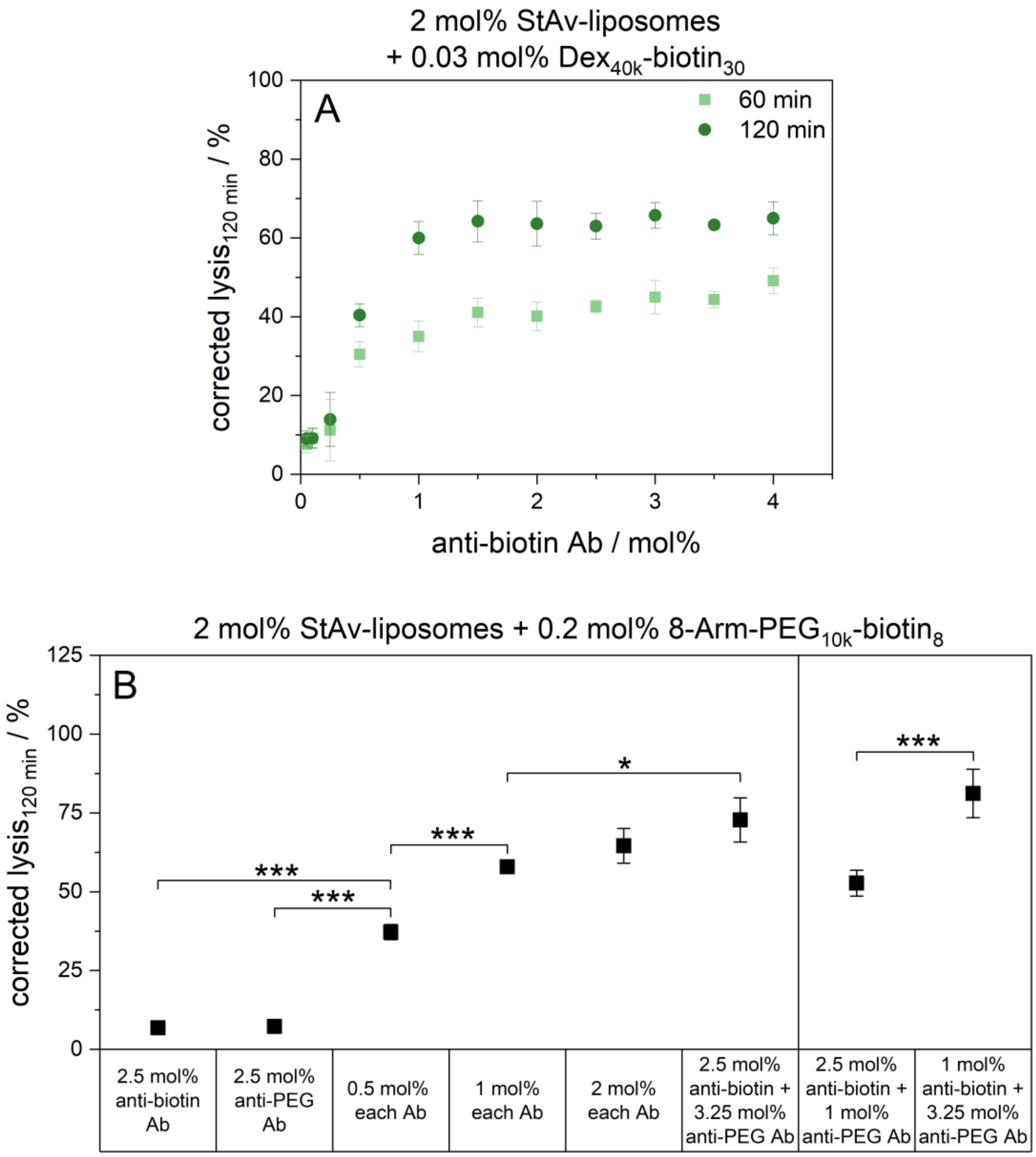

Figure S8: Study of various antibody concentrations and combinations to trigger complement lysis of StAv-liposomes StAv11 (A) or StAv12 (B) in a homogeneous complement assay using **(A)** Dex$_{40k}$-biotin$_{30}$ or **(B)** 8-Arm-PEG$_{10k}$-biotin$_{8}$ as trigger entity. StAv-liposomes and polymer conjugates were incubated for 2 h at RT and 300 rpm. Anti-biotin and anti-PEG antibodies were added, and the samples were further incubated for 1 h at RT and 300 rpm. 5 vol% human serum was used as complement source (IRS45270). Fluorescence measurements were carried out for 120 min at 37 °C in aS or iaS (negative control). The liposome samples were lysed after the measurement by addition of 30 mM OG and incubation for 15 min at RT and 300 rpm. Fluorescence intensities were corrected for the negative control (iaS) and normalized to the

corrected fluorescence of lysed liposomes. λ_{ex} = 565(8) nm and λ_{em} = 585(8) nm; gain 150. T = 37 °C. n = 3.

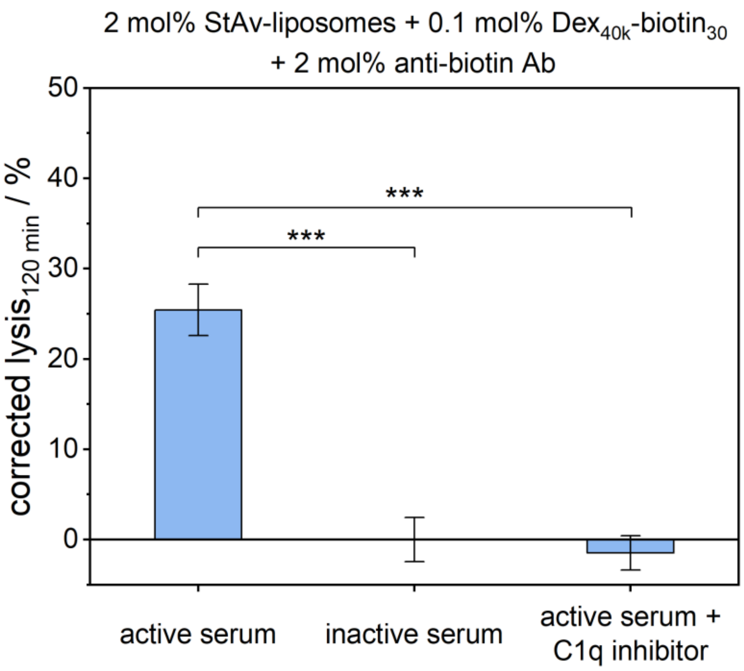

Figure S9: Study of pathway-specific lysis of Dex_{40k}-$biotin_{30}$-mediated complement lysis. StAv-liposomes (StAv14) and 0.1 mol% Dex_{40k}-$biotin_{30}$ were incubated for 2 h at RT and 300 rpm. Anti-biotin antibodies (2 mol%) were added and the samples further incubated for 1 h at RT and 300 rpm. 5 vol% human serum were used per well (IRS45270). Fluorescence measurements were carried out for 120 min at 37 °C in active serum (aS), active serum + C1q inhibitor (25 µg/mL anti-C1q Ab) and inactive serum (iaS). The liposome samples were lysed after the measurement by addition of 30 mM OG and incubation for 15 min at RT and 300 rpm. Fluorescence intensities were corrected for the negative control (iaS) and normalized to the corrected fluorescence of lysed liposomes. λ_{ex} = 565(8) nm and λ_{em} = 585(8) nm; gain = 150; measurement from the top. T = 37 °C. n = 3.

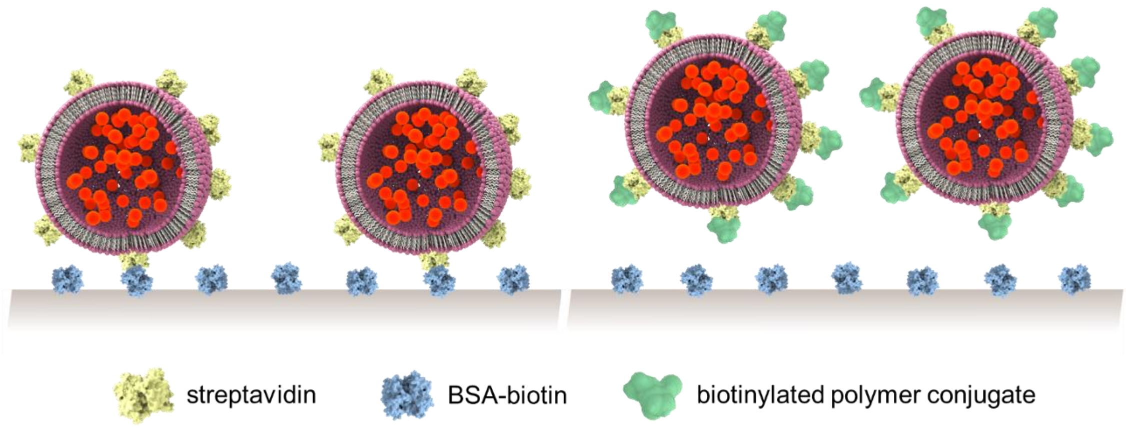

Figure S10: Schematic of the competitive binding assay of StAv-liposomes and biotinylated polymer conjugates (dextran-biotin, 8-Arm-PEG-biotin or BSA-biotin). StAv-liposomes bind to immobilized BSA-biotin (left) and generate a fluorescence signal after washing and lysis with a detergent. In contrast, biotinylated polymer conjugates bind to StAv and inhibit liposome binding to BSA-biotin (right), thereby preventing immobilization and allowing the liposomes to be washed away.

Table S6: DOS, coupling efficiency and EC_{50} values of biotinylated polymer conjugates. DOS values were determined by NMR measurements, and coupling efficiency was calculated from that. EC_{50} values were determined in a competitive binding assay to immobilized BSA-biotin to assess the biotinylation degree and accessibility (Figs. S11-12). Biotinylated polymer conjugates and StAv-liposomes (StAv11) were added to a Nunc MaxiSorp high binding MTP coated with 2 µg/mL BSA-biotin, which was blocked with 1 w/v% BSA in PBS-T and incubated for 3 h at RT and 300 rpm. The plate was washed thrice with HSS (150 µL each) and the remaining liposomes were lysed by addition of 30 mM OG in double dist. H_2O (100 µL, 10 min inc., RT, 300 rpm). λ_{ex} = 560(10) nm and λ_{em} = 585(10) nm, gain 150 or 100. n = 3.

Polymer conjugate	DOS (biotin per glucose unit)	Coupling efficiency	EC_{50} / nM
Dex_{10k}-$biotin_5$	0.11	45%	1.49±0.08
Dex_{10k}-$biotin_{11}$	0.26	52%	1.65±0.11
Dex_{10k}-$biotin_{18}$	0.42	55%	1.30±0.04
Dex_{10k}-$biotin_{24}$	0.56	55%	1.1±2.9
Dex_{40k}-$biotin_{17}$	0.11	42%	0.63±0.03
Dex_{40k}-$biotin_{47}$	0.30	59%	0.68±0.09
Dex_{40k}-$biotin_{77}$	0.49	65%	0.50±0.03
Dex_{40k}-$biotin_{133}$	0.83	83%	8.48±0.28
8-Arm-PEG_{10k}-$biotin_8$	-	108%	1.00±0.04
8-Arm-PEG_{20k}-$biotin_7$	-	89%	0.76±0.08
8-Arm-PEG_{40k}-$biotin_6$	-	75%	0.37±0.05
BSA-$biotin_9$	-	-	1.05±0.05

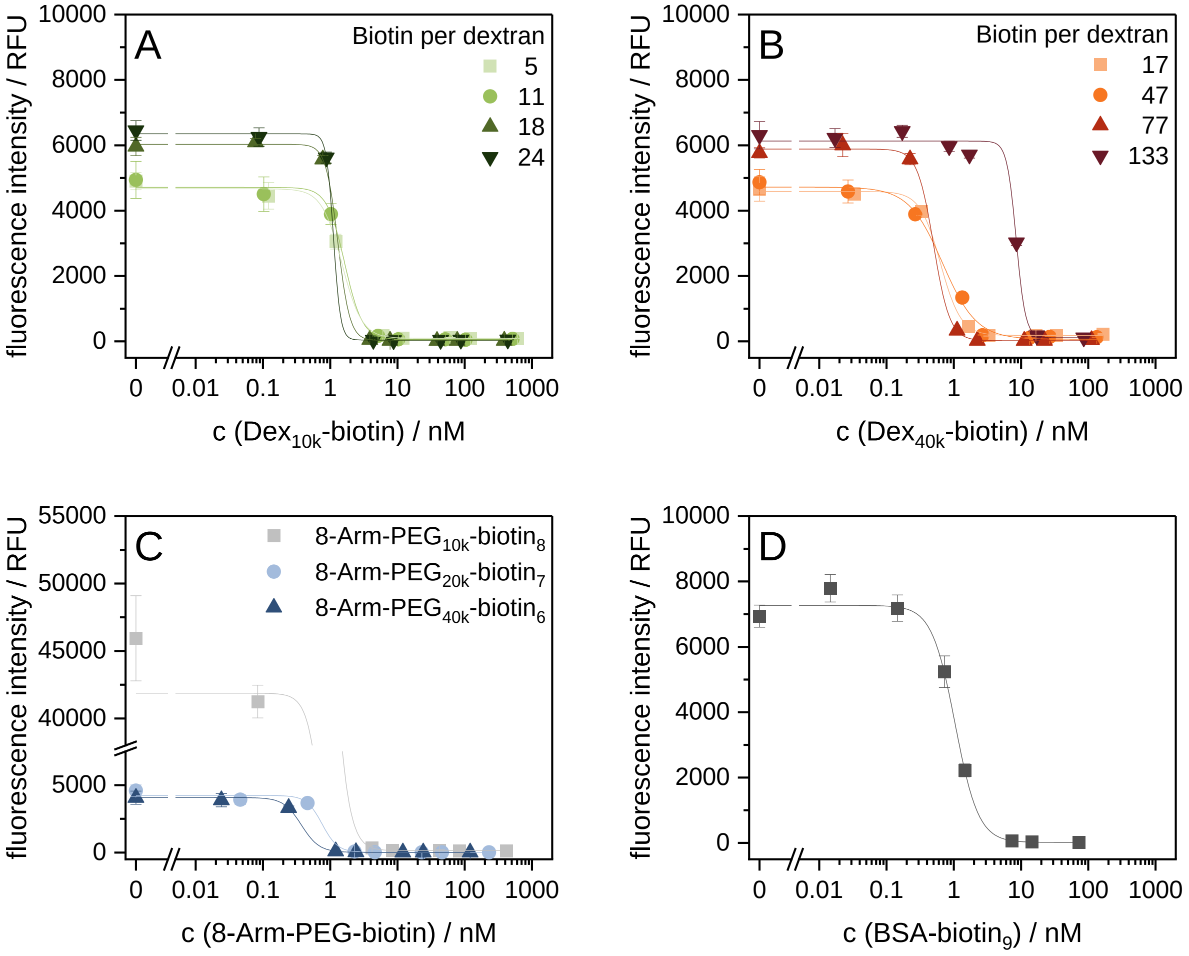

Figure S11: Competitive binding assay of biotinylated polymer conjugates (Dex_{10k}-biotin **(A)**, Dex_{40k}-biotin **(B)**, 8-Arm-PEG-biotin **(C)**, BSA-$biotin_9$ **(D)**) to immobilized BSA-biotin to assess their biotinylation degree and accessibility. Biotinylated polymer conjugates and StAv-liposomes (StAv11) were added to a Nunc MaxiSorp high binding MTP coated with 2 µg/mL BSA-biotin, which was blocked with 1 w/v% BSA in PBS-T, and incubated for 3 h at RT and 300 rpm. The plate was washed thrice with HSS (150 µL each) and the remaining liposomes were lysed by addition of 30 mM OG in double dist. H_2O (100 µL, 10 min inc., RT, 300 rpm). λ_{ex} = 560(10) nm and λ_{em} = 585(10) nm, gain 150 or 100. n = 3.

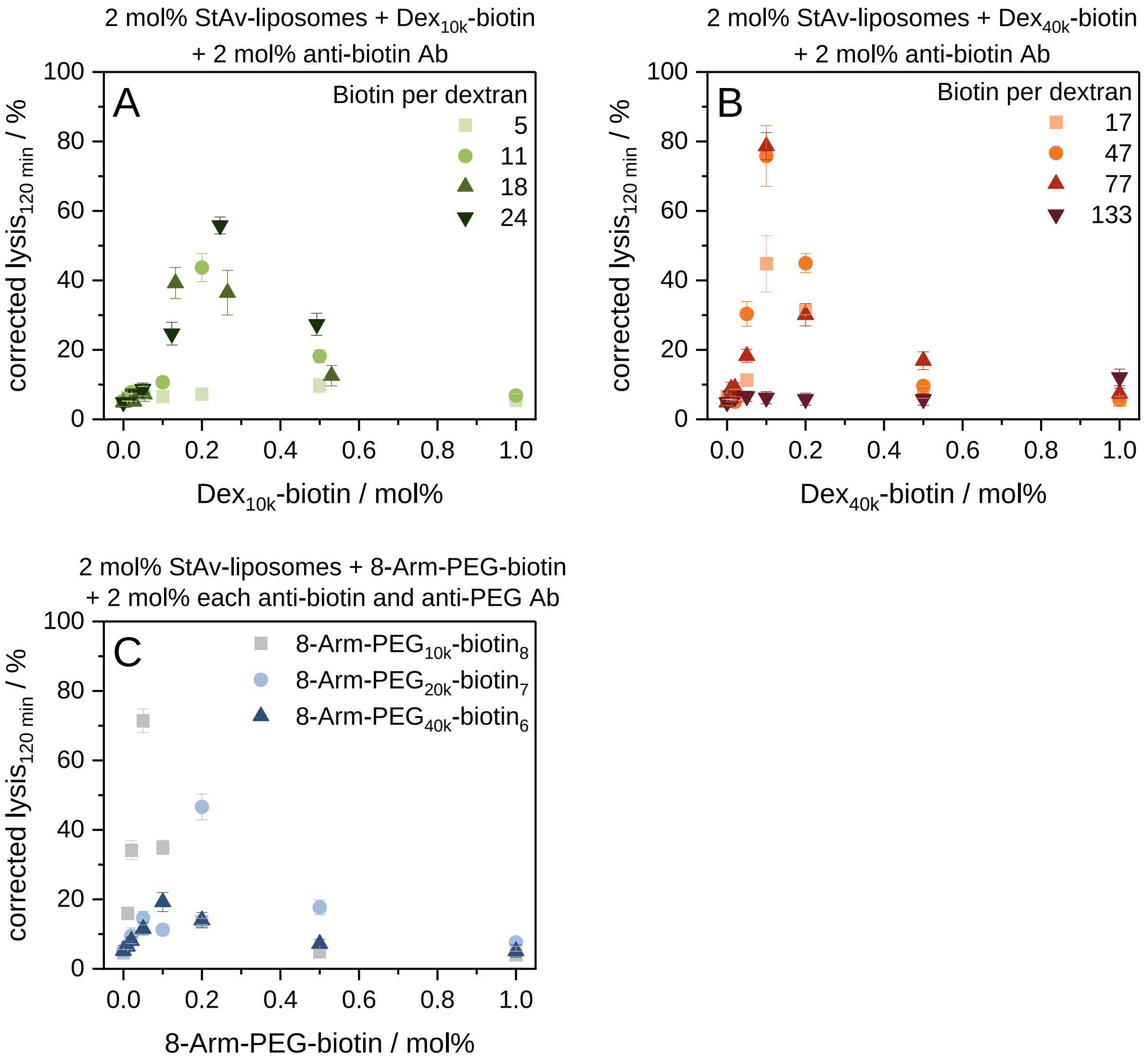

Figure S12: Corrected lysis of StAv-liposomes (StAv12) in complement assays investigating various biotinylated trigger entities (Dex_{10k}-biotin **(A)**, Dex_{40k}-biotin **(B)**, 8-Arm-PEG-biotin **(C)**) with different molecular weights and biotinylation degrees in a titration using an anti-biotin Ab and an anti-PEG Ab as complement trigger. StAv-liposomes (StAv12) and biotinylated polymer conjugates were incubated for 2 h at RT and 300 rpm. Antibodies were added and the samples were further incubated for 1 h at RT and 300 rpm. 5 vol% human serum was used as complement source (IRS45270). Fluorescence measurements were carried out for 120 min at 37 °C in aS or iaS. The liposome samples were lysed after the measurement by addition of 30 mM OG and incubation for 15 min at RT and 300 rpm. The aS samples were corrected for

the negative control (iaS) and normalized to the corrected fluorescence of lysed liposomes. λ_{ex} = 565(8) nm and λ_{em} = 585(8) nm; gain 150. T = 37 °C. n = 3.

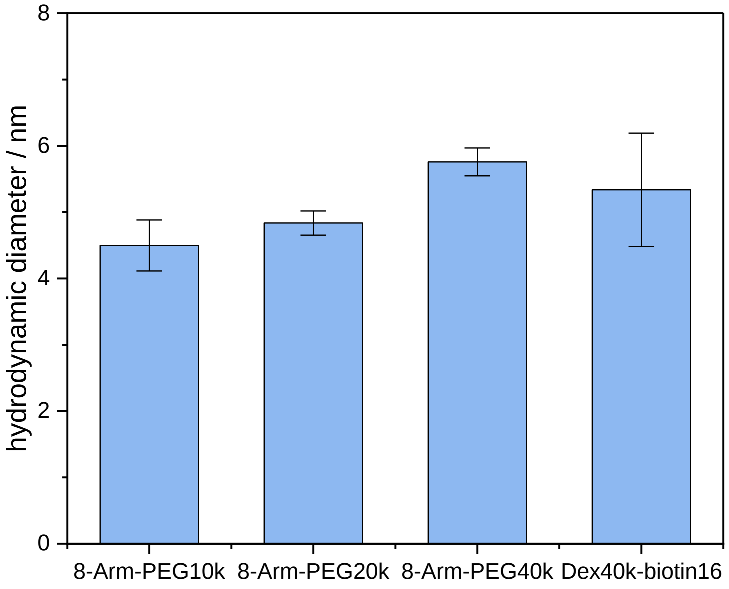

Figure S13: Hydrodynamic diameters of different polymer conjugates. Data were recorded with a Malvern Zetasizer Nano and weighted by number average. Samples were prepared in MilliQ water and filtered through 0.2 µm PES filters. n = 3.

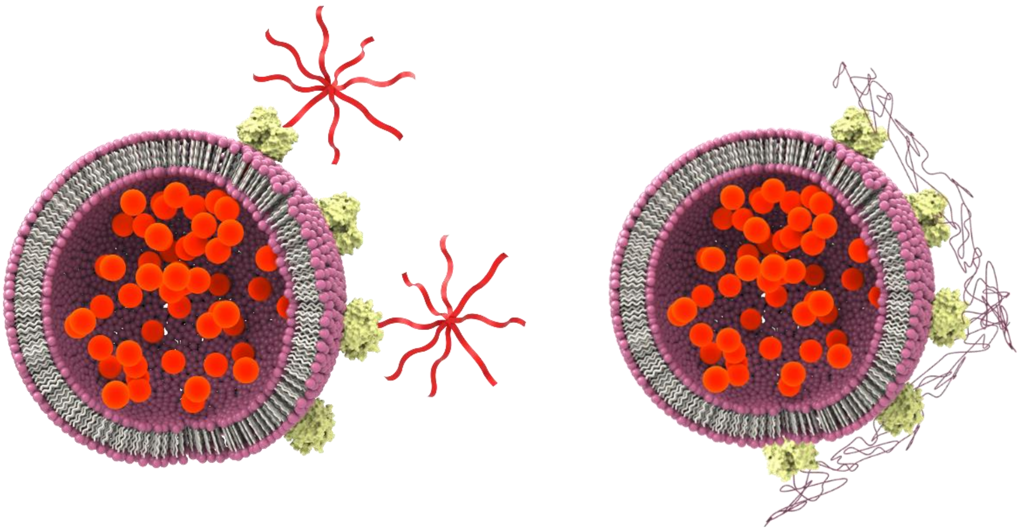

Figure S14: Schematic of the hypothesized structures of 8-Arm-PEG-biotin (left) and dextran-biotin (right) when bound to the liposome surface. The 8-Arm-PEG-biotin polymer conjugate has only terminal biotin modifications and therefore retains its range of motion. Dextran-biotin is modified along the chain, allowing the dextran to unfold and bind multiple streptavidin molecules, thereby spreading across the liposome surface.

Table S7: Hydrodynamic diameter, PDI and max. complement lysis values of Stav-liposomes (StAv13) along with various concentrations of Dex_{40k}-$biotin_{30}$ determined by DLS or in a complement assay. StAv-liposomes and Dex_{40k}-$biotin_{30}$ were incubated for 2 h at RT and 300 rpm. Anti-biotin antibodies were added and the samples were further incubated for 1 h at RT and 300 rpm. 5 vol% human serum was used as complement source (IRS45270). Fluorescence measurements were carried out for 120 min at 37 °C in aS. The aS samples were corrected for the negative control (iaS) and normalized to the corrected fluorescence of lysed liposomes. λ_{ex} = 565(8) nm and λ_{em} = 585(8) nm; gain 150. T = 37 °C. n = 3.

mol% Dex_{40k}-$biotin_{30}$	hydrodynamic diameter / nm	PDI	complement lysis / %
0	136 ± 2	0.16 ± 0.02	8 ± 3
0.01	140 ± 1	0.17 ± 0.02	10 ± 2
0.02	145 ± 2	0.15 ± 0.03	7 ± 2
0.05	204 ± 9	0.38 ± 0.01	8 ± 1
0.1	204 ± 3	0.40 ± 0.06	42 ± 4
0.2	170 ± 1	0.25 ± 0.01	26 ± 5
0.5	160 ± 1	0.25 ± 0.01	11 ± 1
1.0	148 ± 3	0.18 ± 0.01	9 ± 1

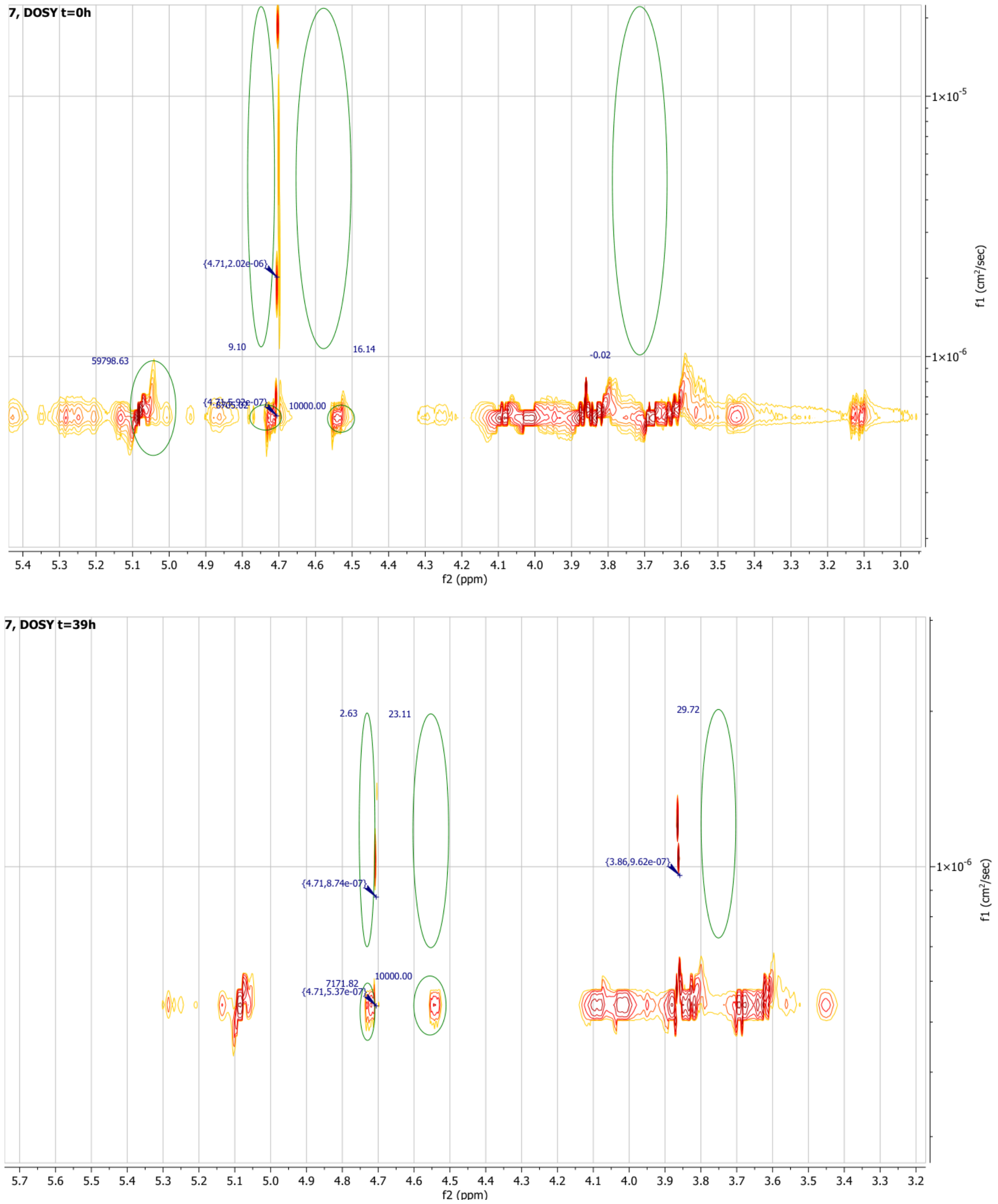

Figure S15: Results of an isothermal stress test of a Dex$_{40k}$-biotin$_{26}$ using a DOSY-^{1}H-NMR. After 39 h at 45°C, no changes to the spectrum could be recorded. Biotin peaks (4.5 and 4.7 ppm) are compared to a background signal (3.75 ppm) and are of similar magnitude. Hydrolysis does either not take place or can be safely estimated to be < 1%. The polymer conjugates were therefore viewed as stable for the duration of this study.

NMR spectra of dextran- and 8-Arm-PEG-based polymer conjugates

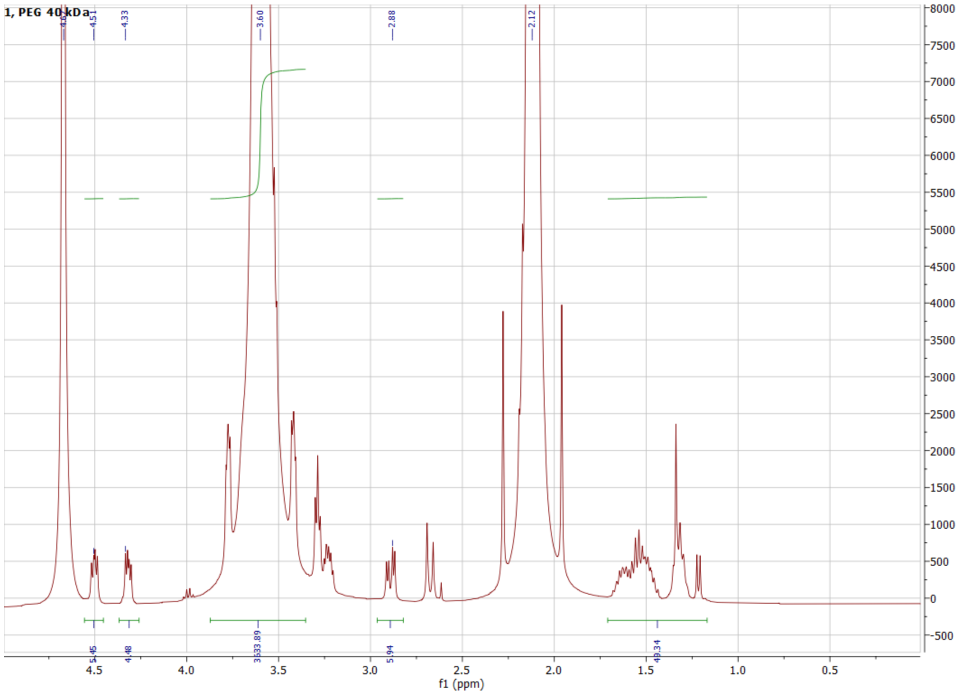

Figure S16: ^{1}H-NMR spectrum of an 8-Arm-PEG$_{40k}$-biotin$_{6}$ recorded with a Bruker Avance Neo 500 MHz in D_2O

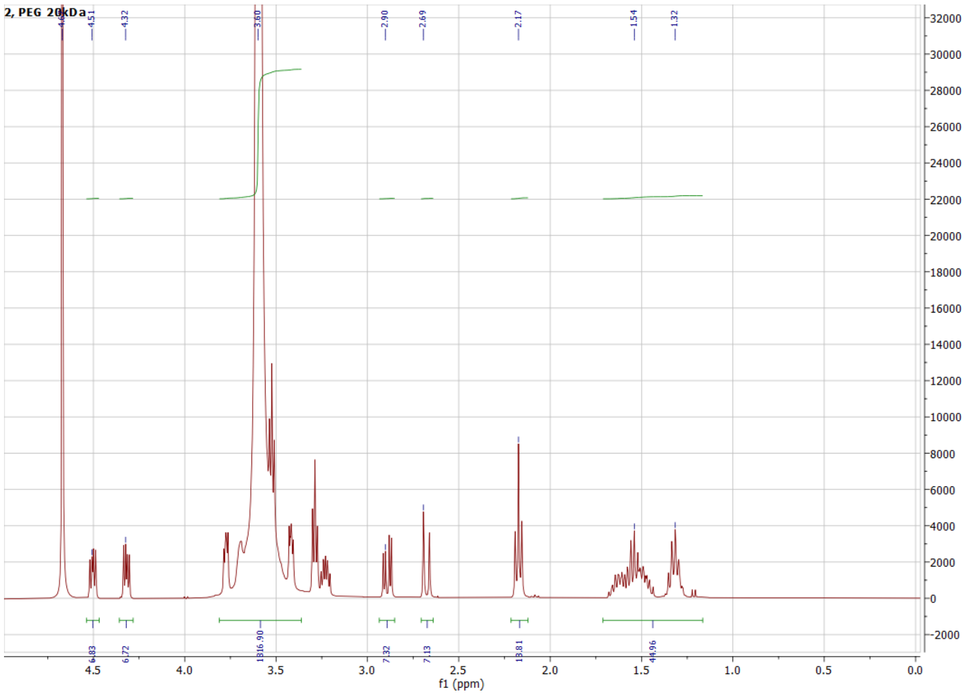

Figure S17: ^{1}H-NMR spectrum of an 8-Arm-PEG$_{20k}$-biotin$_{7}$ recorded with a Bruker Avance Neo 500 MHz in D_2O.

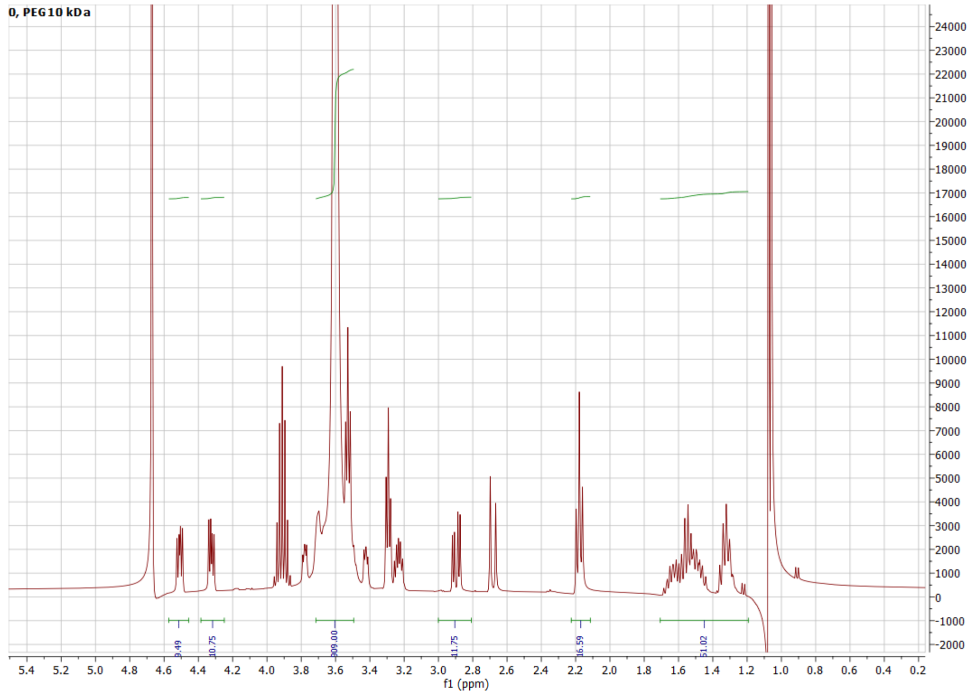

Figure S18: ^{1}H-NMR spectrum of an 8-Arm-PEG$_{10k}$-biotin$_{8}$ recorded with a Bruker Avance Neo 500 MHz in D_2O.

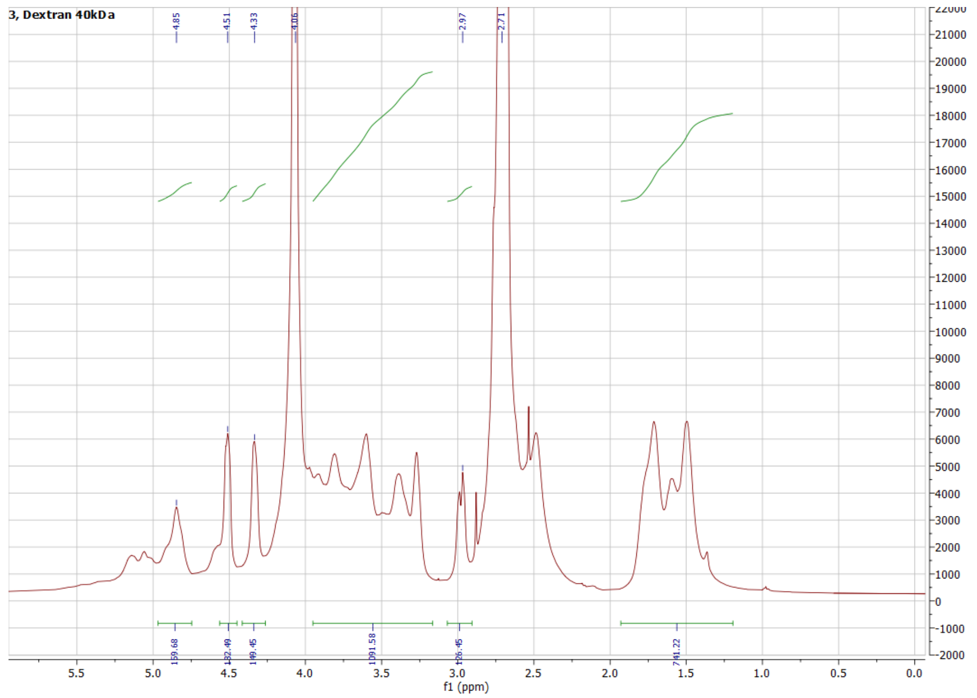

Figure S19: ^{1}H-NMR spectrum of a dextran$_{40k}$-biotin$_{133}$ recorded with a Bruker Avance Neo 500 MHz in DMSO-d_6.

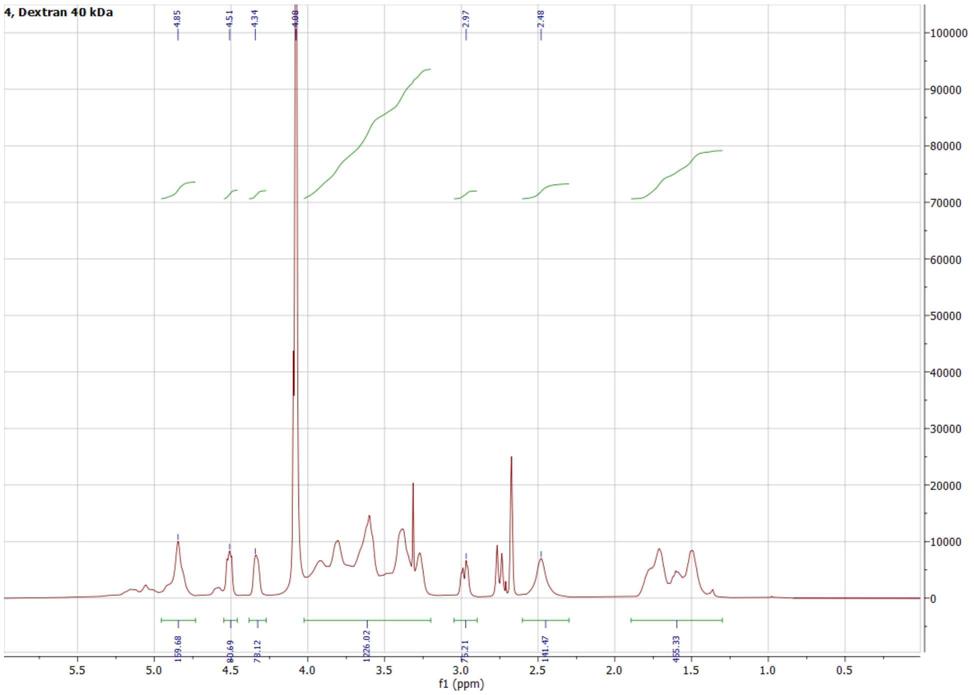

Figure S20: ^{1}H-NMR spectrum of dextran$_{40k}$-biotin$_{77}$ recorded with a Bruker Avance Neo 500 MHz in DMSO-d$_6$.

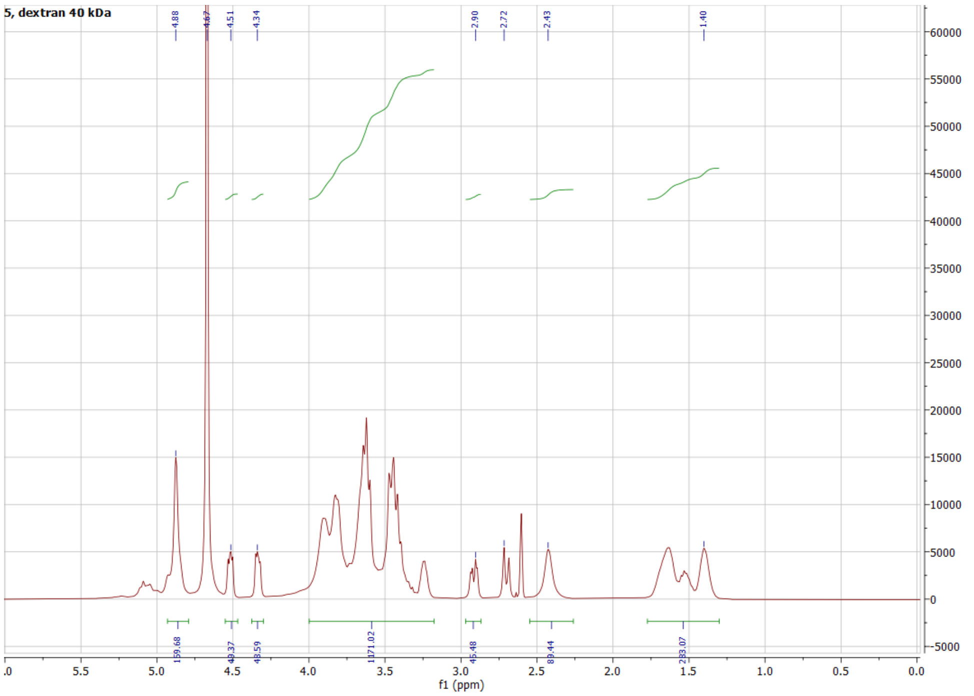

Figure S21: ^{1}H-NMR spectrum of dextran$_{40k}$-biotin$_{47}$ recorded with a Bruker Avance Neo 500 MHz in D_2O.

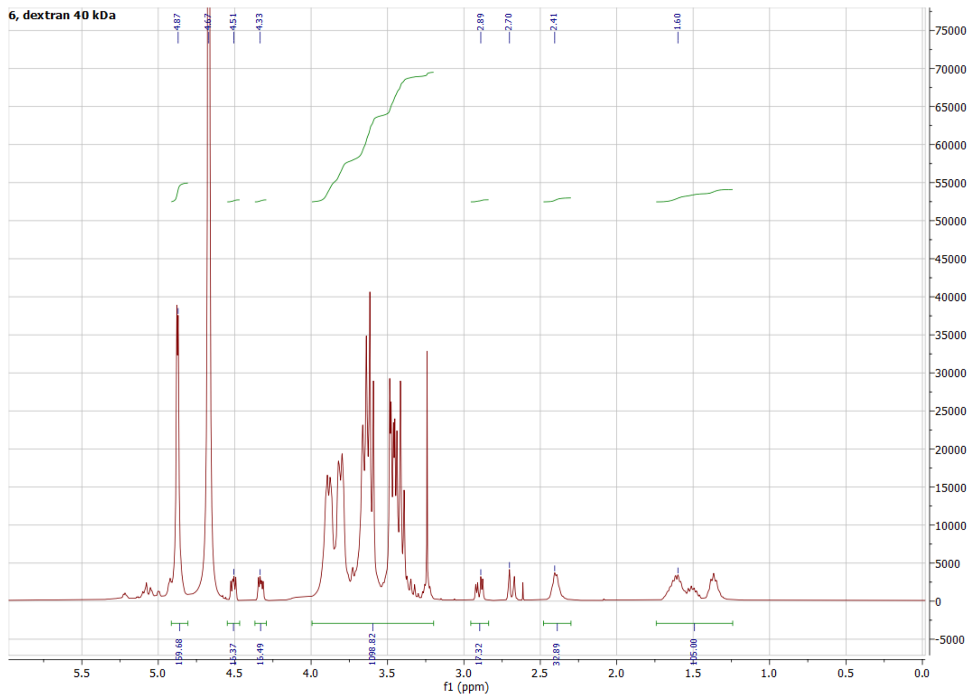

Figure S22: ^{1}H-NMR spectrum of dextran$_{40k}$-biotin$_{17}$ recorded with a Bruker Avance Neo 500 MHz in D_2O.

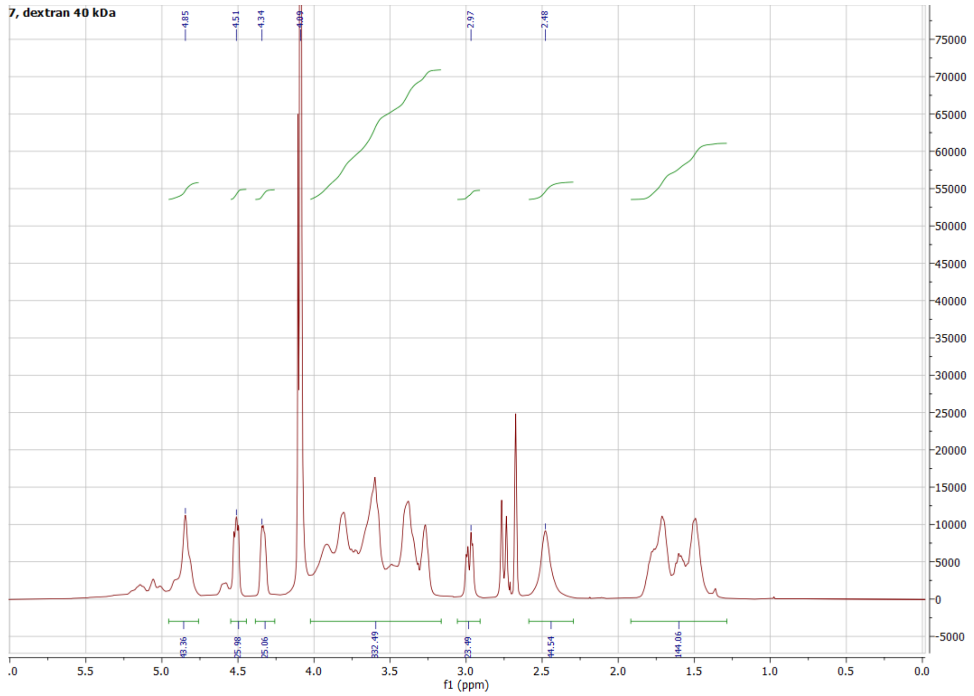

Figure S23: ^{1}H-NMR spectrum of dextran$_{10k}$-biotin$_{24}$ recorded with a Bruker Avance Neo 500 MHz in DMSO-d_6.

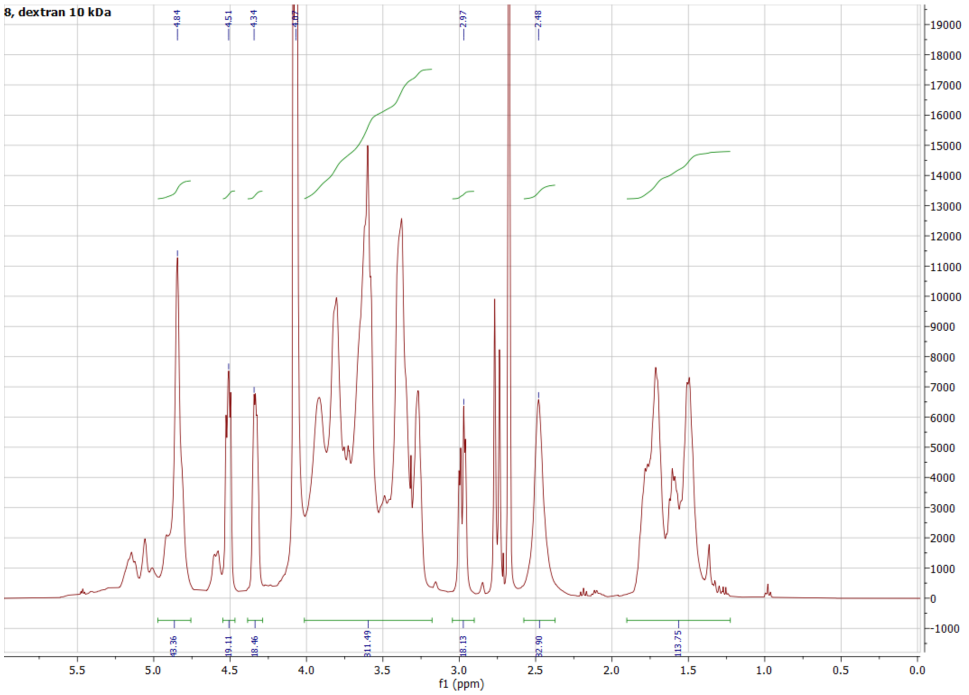

Figure S24: ^{1}H-NMR spectrum of dextran$_{10k}$-biotin$_{18}$ recorded with a Bruker Avance Neo 500 MHz in DMSO-d$_6$.

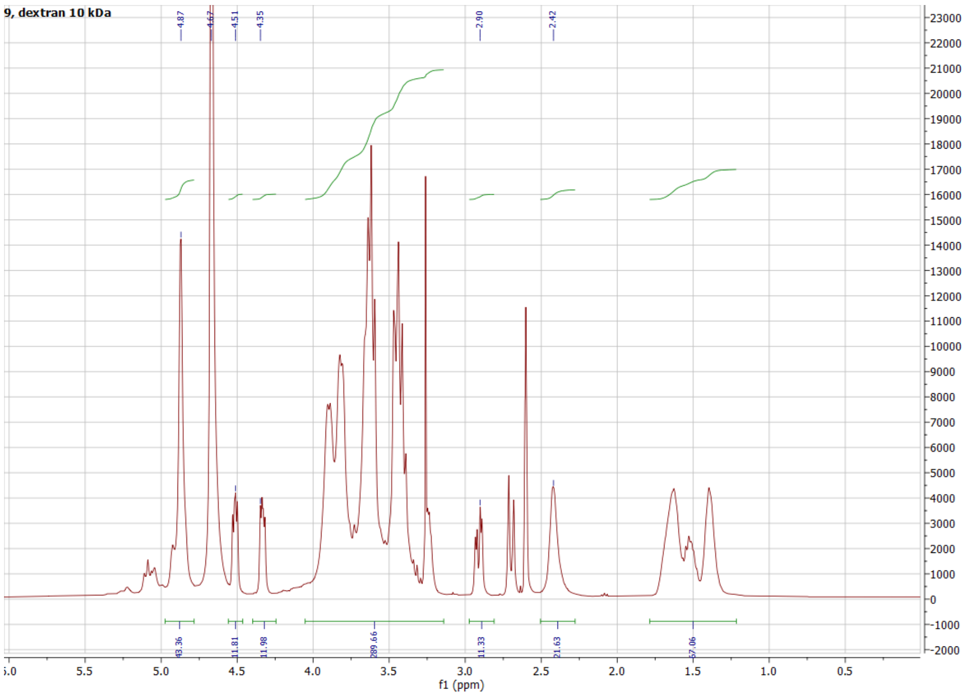

Figure S25: ^{1}H-NMR spectrum of dextran$_{10k}$-biotin$_{11}$ recorded with a Bruker Avance Neo 500 MHz in D_2O.

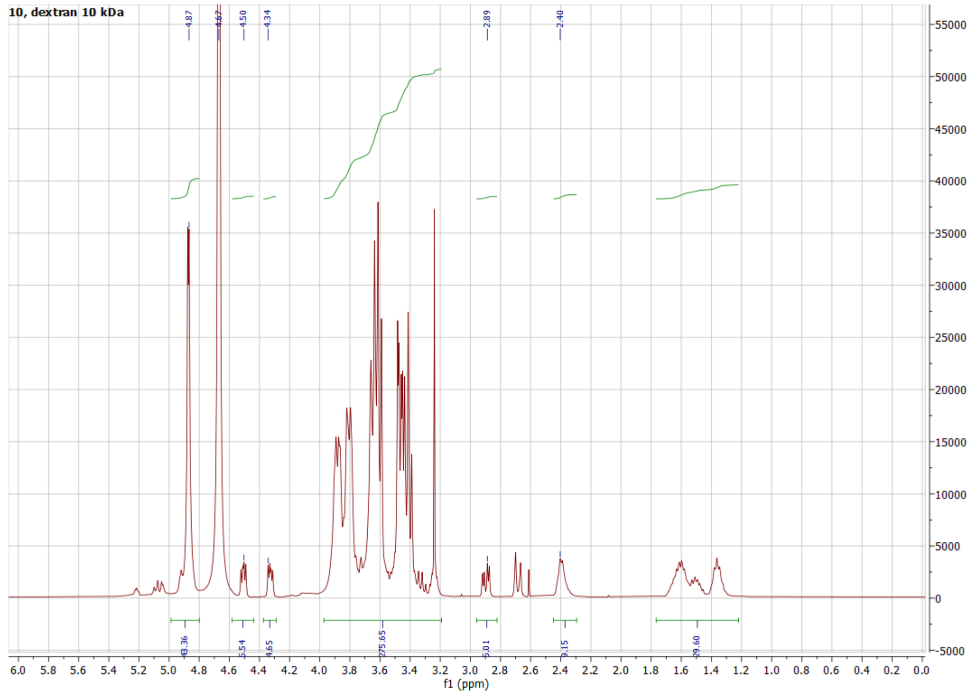

Figure S26: ^{1}H-NMR spectrum of dextran$_{10k}$-biotin$_5$ recorded with a Bruker Avance Neo 500 MHz in D_2O.

References

1. Hoecherl K, Streif S, Spitzenberg C, Rink S, Behrent A, Holzhausen F, Griesche C, Rogoll C, Foedlmeier M, Gebhard A, Kulikowski K, Schaefer N, Pauly D, Baeumner AJ. A homogeneous immunoassay technology based on liposomes and the complement system enables one-step, no-wash, rapid diagnostics directly in serum. Anal Bioanal Chem. 2025;417:3257–73. doi:10.1007/s00216-025-05882-4.
